# MTOR signaling regulates the development of airway mucous cell metaplasia associated with severe asthma

**DOI:** 10.1172/jci.insight.187904

**Published:** 2025-05-29

**Authors:** Katrina M. Kudrna, Luis F. Vilches, Evan M. Eilers, Shailendra K. Maurya, Steven L. Brody, Amjad Horani, Kristina L. Bailey, Todd A. Wyatt, John D. Dickinson

**Affiliations:** 1 Department of Internal Medicine, Division of Pulmonary, Critical Care, & Sleep, University of Nebraska Medical Center, Omaha, Nebraska, USA.; 2 Department of Medicine, Division of Pulmonary and Critical Care, Washington University, St. Louis, Missouri, USA.; 3 Washington University School of Medicine, Division of Pediatric Allergy, Immunology, and Pulmonary Medicine, St. Louis, Missouri, USA.; 4 Department of Veterans Affairs Nebraska-Western Iowa Health Care System, Omaha, Nebraska, USA.; 5 Department of Environmental, Agricultural and Occupational Health, College of Public Health, University of Nebraska Medical Center, Omaha, Nebraska, USA.

**Keywords:** Cell biology, Pulmonology, Asthma, Autophagy, Cell migration/adhesion

## Abstract

In asthma, airway epithelial remodeling is characterized by aberrant goblet cell metaplastic differentiation accompanied by epithelial cell hyperplasia and hypertrophy. These pathologic features in severe asthma indicate a loss of control of proliferation, cell size, differentiation, and migration. MTOR is a highly conserved pathway that regulates protein synthesis, cell size, and proliferation. We hypothesized that the balance between MTOR and autophagy regulates mucous cell metaplasia. Airways from individuals with severe asthma showed increased MTOR signaling by RPS6 phosphorylation, which was reproduced using an IL-13–activated model of primary human airway epithelial cells (hAEC). MTOR inhibition by rapamycin led to a decrease of IL-13–mediated cell hypertrophy, hyperplasia, and MUC5AC mucous metaplasia. BrdU labeling during IL-13–induced mucous metaplasia confirmed that MTOR was associated with increased basal-to-apical hAEC migration. MTOR activation by genetic deletion of Tsc2 in cultured mouse AECs increased IL-13–mediated hyperplasia, hypertrophy, and mucous metaplasia. Transcriptomic analysis of IL-13–stimulated hAEC identified MTOR-dependent expression of genes associated with epithelial migration and cytoskeletal organization. In summary, these findings point to IL-13–dependent and –independent roles of MTOR signaling in the development of pathogenic epithelial changes contributing to airway obstruction in severe asthma.

## Abstract 2

Severe asthma is associated with remodeling of the epithelium leading to airway obstruction. mTOR signaling is critical to this maladaptive epithelial response.

## Graphical abstract

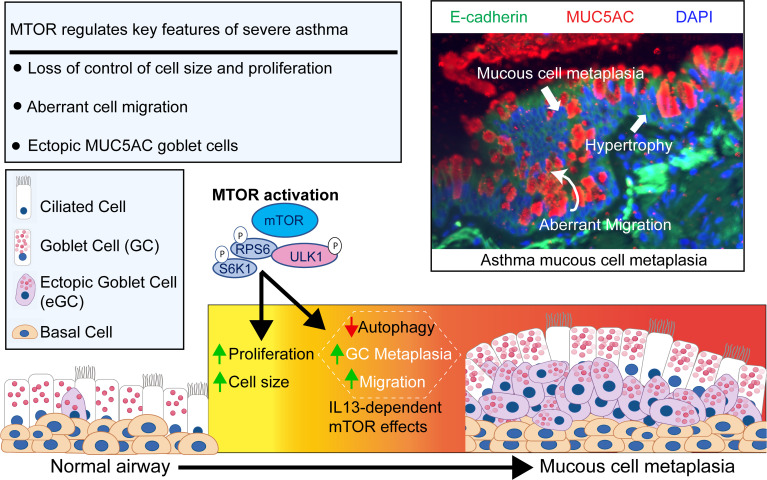

## Introduction

Cells tightly regulate proliferation, size, and differentiation in response to nutrient availability, and inflammatory or infectious cues. Consequently, airway epithelial cells must adapt to external signals for specialized functions. Much is known about the control of the identity, localization, and function of individual airway epithelial cells in the healthy airway (1–3). However, during inflammation, injury, and repair, the maintenance of cell classification and function is perturbed. In response to type 2 airway inflammation, basal cells and secretory cells differentiate to large mucous-secreting cells that are tightly packed with mucin granules that are morphologically termed goblet cells (GC). Histologic studies from autopsy series (4) from fatal asthma attacks and endobronchial biopsies from mild and severe asthmatics (5, 6) have revealed loss of control of epithelial cell size, migration, and proliferation along with GC metaplasia. Increased airway wall thickness collectively is associated with mucous plugging, contributing to airway obstruction and loss of lung function (7). Type 2 cytokines, such as IL-13, play an important role in the development of airway obstruction driving epithelial hyperplasia, GC hypertrophy, mucous hypersecretion, and loss of polarity with aberrant migration (8–12). However, airway epithelial cellular migration in the context of type 2 inflammation remains unresolved. Traditional wounding assays have demonstrated conflicting results on the role of IL-13 on migration (8, 9). At a cellular level, the loss of control of epithelial cell size, proliferation, and migration in the context of dynamic exogenous inflammatory signaling associated with severe asthma is incompletely understood.

Mammalian target of rapamycin (MTOR) is a nutrient-sensing signaling protein complex that regulates cell size, metabolism, migration, and proliferation while inhibiting autophagy (13, 14). The MTOR pathway is a highly conserved serine-threonine kinase signaling complex located at the hub of a network. Two distinct signaling protein complexes (MTORC1 and MTORC2) exist with differing cofactors and phosphorylation targets. MTORC1 phosphorylates S6K1 and ribosomal S6 (RPS6) leading to increased protein synthesis and anabolic metabolism required for cell growth and proliferation (15–17). MTOR also inhibits autophagy through phosphorylation of unc-51 like autophagy activating kinase 1 (ULK1) and autophagy-related protein 13 (ATG13) (18–21). Macroautophagy (referred to here as autophagy) provides a counterbalance to these metabolic demands through bulk protein degradation-recycling proteins to amino acids for new synthesis (22–24).

We and others have reported that autophagy proteins are increased in the airway epithelium during cytokine-mediated mucous metaplasia in vitro (12, 25) and in diseased human airway samples (26). However, we have not been able to reconcile the profound morphologic differences driven by mitogenic effects of cytokines such as IL-13 on the epithelial hyperplasia, hypertrophy, and mucous cell metaplasia with increased autophagy signaling, which is catabolic in nature. Although autophagy is commonly thought to be activated in airway epithelial mucous cell metaplasia, the profound morphologic changes and increased protein production point to a more complex situation in which the balance of MTOR and autophagy may regulate key biologic processes critical to the development, persistence, and resolution of airway disease. Here, we propose a potentially new conceptual framework of mucous cell metaplasia in which the temporal balance of MTOR and autophagy control features of pathogenic mucous cell metaplasia. IL-13–mediated MTOR activation drives loss of control of airway epithelial cell proliferation, hypertrophy, and migration, contributing to airway wall thickening and formation of eGC (eGC), which are key features of mucous cell metaplasia found in severe asthma.

## Results

### Mucous cell metaplasia in severe asthma is characterized by the emergence of eGC associated with regions of epithelial hyperplasia and hypertrophy.

**Figure 1:**
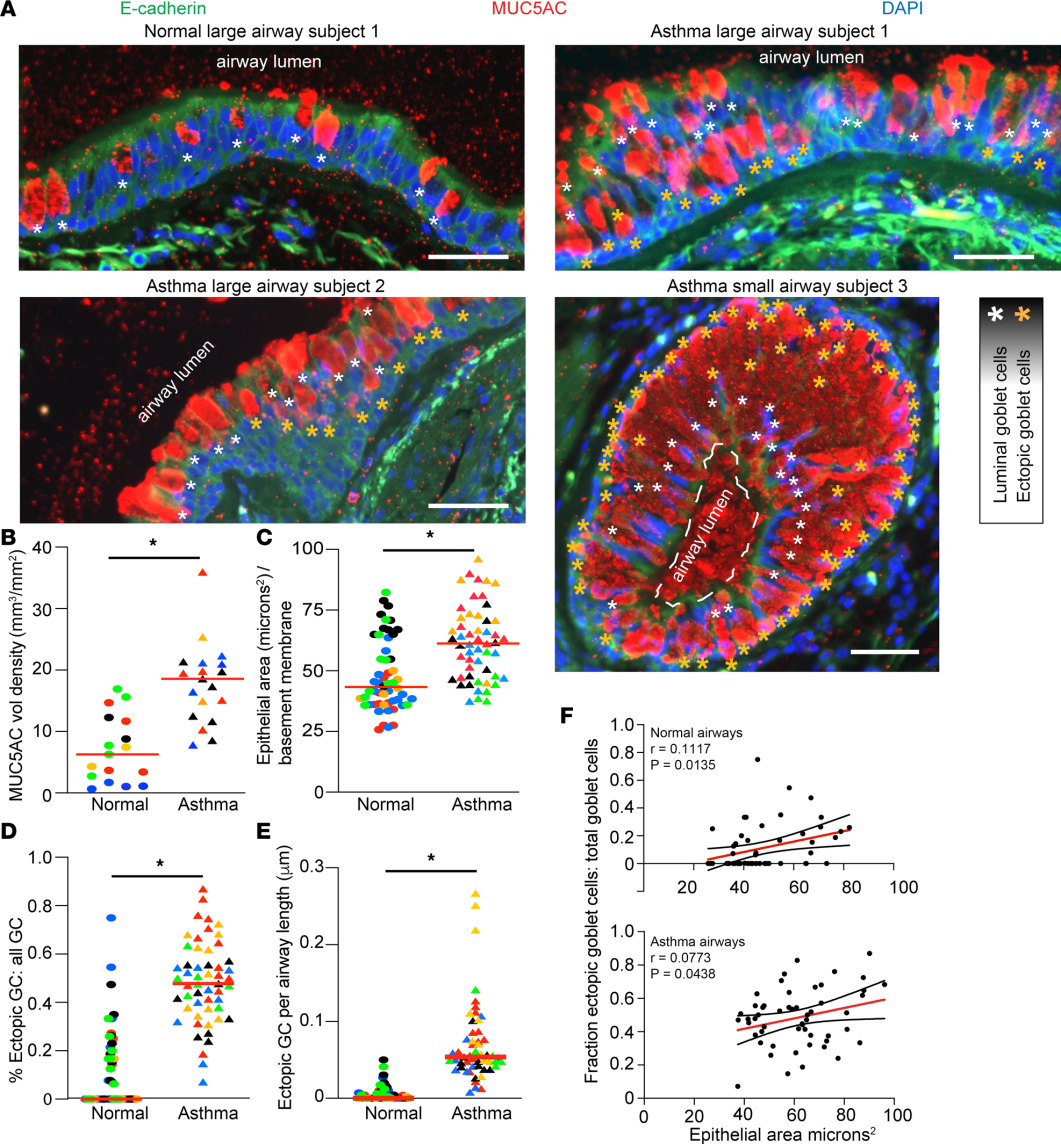
Airway epithelium from severe asthmatics is characterized by mucous metaplasia with ectopic goblet cell distribution. (**A**) Representative airway immunostaining for MUC5AC and E-Cadherin: Large airway section from nonasthmatic control (upper left panel), large airway section from #1 asthmatic sample (upper right panel), large airway section from #2 asthma sample (lower left panel), small airway section from #3 asthmatic sample (lower right panel). White asterisk marks airway luminal surface goblet cells (GC), golden asterisk marks airway ectopic goblet cells (eGC). DAPI for nuclear counter stain. Scale bar: 50 μm. (**B**) Quantification of MUC5AC volume density per airway. Each color represents a unique nonasthma or asthma patient sample. (**C**) The total epithelial area μm^2^ per airway basement membrane segment was determined for nonasthma controls and asthmatic airways. (**D** and **E**) The fraction of ectopic goblet cells (eGC) relative to total goblet cells and total cells along the linear baseline membrane was calculated with *n* = 5 asthmatic airway and 5 normal airway donors with *n* = 8 to 15 distinct images per donor airway. Unpaired *t*-test (2-tailed) for statistical difference with *P < 0.05. (**F**) Simple linear regression comparison of airway epithelial area and fraction eGC relative to total GC. Upper panel is nonasthma controls, and lower panel is asthmatic airways. Mean slope and 95% CI are shown.

In severe asthma, the airway epithelium undergoes remodeling characterized by increased epithelial thickness and mucous cell metaplasia with increased size and number of GC, collectively contributing to airway obstruction (4–6, 27, 28). To identify key pathologic features of the asthmatic airway epithelia, we examined segmental bronchi cross sections that contained a defined lamina propria layer tangential to the epithelium. Airway sections from nonasthma controls contained a pseudostratified epithelial layer with irregularly localized MUC5AC^+^ GC along the luminal surface (Figure 1A). In contrast, airway sections from patients with severe asthma demonstrated significantly increased epithelial thickness, increased numbers of MUC5AC^+^ GC (Figure 1, A–C), and occasional luminal occlusion by a mucous plug in the small airways (<2 mm in diameter) (Figure 1A). Furthermore, the epithelial layer was fundamentally disorganized, characterized by multiple layers of MUC5AC^+^ GC. In both large and small airways, many of the GC were localized deep within the epithelial layer without an outlet to the luminal surface. We termed these morphologically abnormally localized GC “eGC.” To better characterize eGC, we utilized E-cadherin to mark the cell borders of individual cells in the airway cross sections. The fraction of eGC relative to all GC, and total number of eGC per airway segment, were significantly increased in eGC from asthma airways (Figure 1, D and E). eGC were not unique to asthmatic airway sections. Some nonasthma control airway sections also contained scattered eGC, though at a lower frequency. Furthermore, we identified hypertrophic areas of increased epithelial thickness in individuals with severe asthma. These hypertrophied areas contained a higher density of eGC. The fraction of eGC was related to the epithelial thickness of the corresponding airway epithelial segment (Figure 1F). These epithelial hypertrophied areas may reflect regions of high aberrant metaplastic differentiation and migration. We therefore sought to understand the factors that led to these regions of hypertrophy, MUC5AC mucous cell metaplasia, and aberrant migration.

### MTOR signaling is increased in severe asthma airways with mucous cell metaplasia.

**Figure 2:**
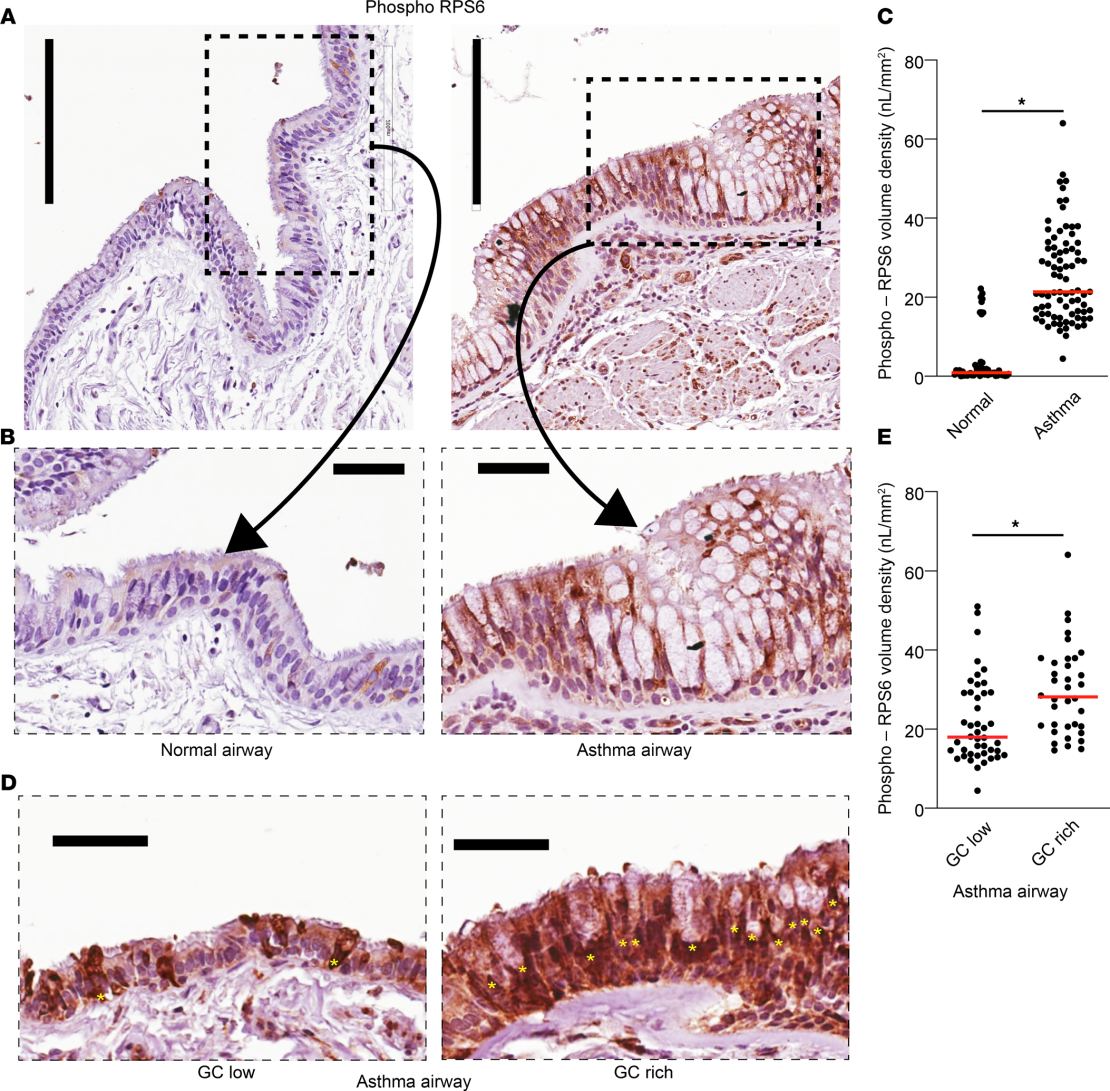
Increased MTOR signaling in asthmatic airway epithelium. (**A**) Representative IHC image of MTOR substrate ribosomal S6 phosphorylated at serine 240/244 (phospho-RPS6). Images from nonasthma controls on the left and asthmatic airway sections on the right. Vertical scale bar: 200 μm. (**B**) Dashed box indicates area of higher magnification. Scale bar: 25 μm. (**C**) Quantification of phospho-RPS6 levels normalized to volume density per airway. (**D**) Representative IHC image of phospho-RPS6 from severe asthma airways classified based on morphologic identification of goblet cells (GC) as either GC low or GC rich (estimated ≥ 50% GC). Yellow asterisks mark GC. Scale bar: 25 μm. (**E**) Quantification of phospho-RPS6 staining in GC low versus GC rich regions. *N* = 5 nonasthma controls and 5 asthmatic airway donors. *N* = 10–15 images per donor. Unpaired *t*-test (2-tailed) for statistical difference with *P < 0.05.

Metaplastic airway epithelium in asthma is characterized by many of the central features of MTOR activation: increased epithelial cell hyperplasia and cellular hypertrophy. We examined the airway sections from nonasthma controls and individuals with severe asthma for MTOR activation using phosphorylated serine 240/244 (phospho-RPS6), which regulates key MTOR-dependent signaling pathways in cell proliferation, cell size, and protein synthesis (29). We found a significant increase in phospho-RPS6 staining in asthma epithelial sections compared with controls (Figure 2, A–C). In contrast, the phospho-RPS6 staining was scattered in individual cells in the nonasthma airways. This finding indicates that MTOR signaling is activated in the airway epithelium of severe asthmatics. We further subclassified severe asthma airways into 2 categories by the morphologic identification of GC. Airway sections with < 50% GC were classified as low GC airways. Sections with estimated GC > 50% were classified as GC rich. Phospho-RBS6 staining was significantly higher in the GC-rich asthma airway sections than the GC-low asthma airways (Figure 2, D and E). This suggests that MTOR signaling may be associated with mucous cell metaplasia.

**Figure 3:**
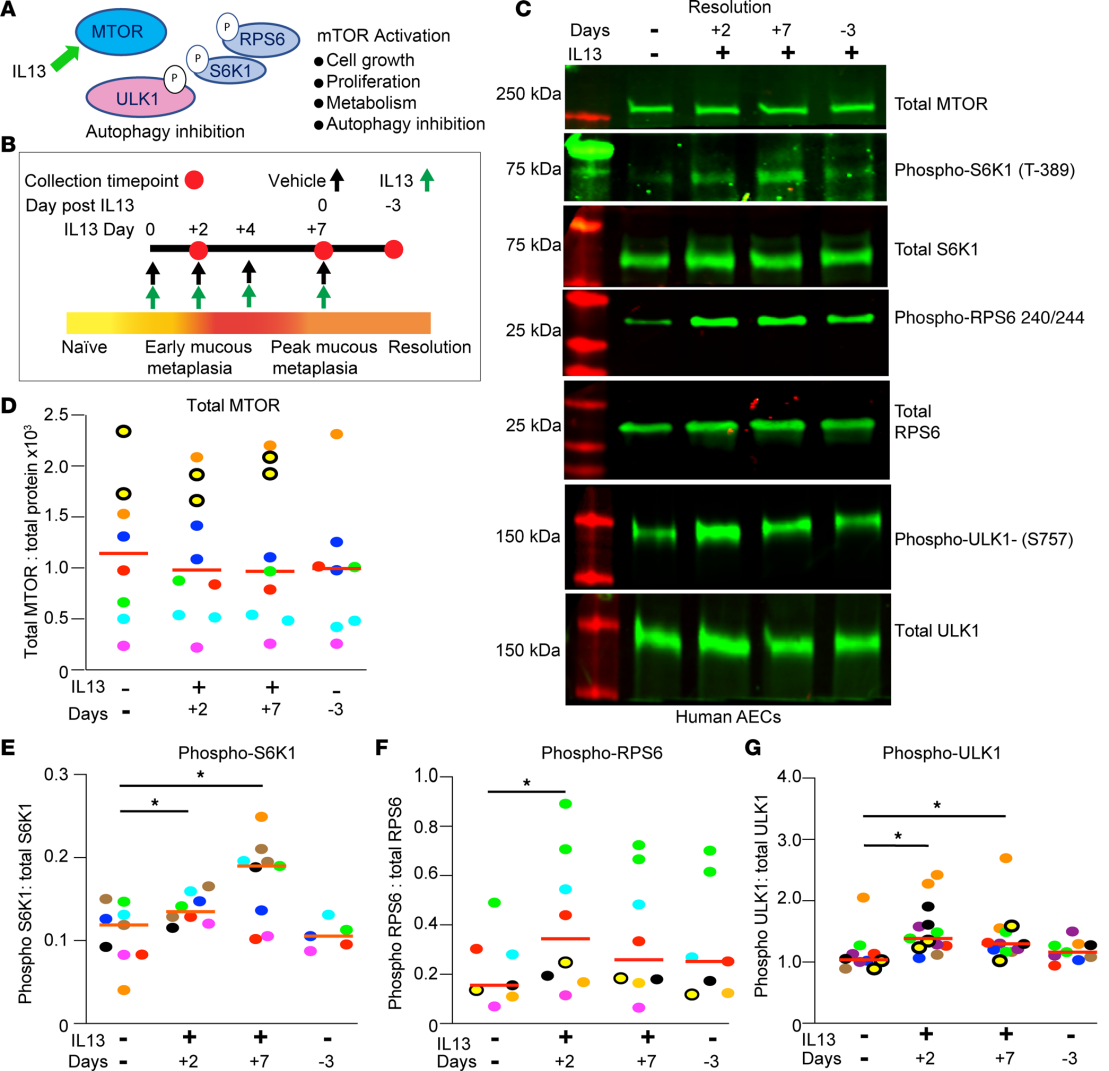
IL-13 increases MTOR substrate phosphorylation. (**A**) MTOR signaling regulates both MTOR and autophagy by protein phosphorylation. (**B**) Schematic for IL-13–mediated mucous metaplasia (IL-13, 10 ng/mL) in human airway epithelial cells (hAEC) and resolution according to time points after withdrawal of IL-13. (**C**) Representative immunoblots for MTOR levels and total and phosphorylated RPS6 (S240/244), S6K1 (T389), and ULK1 (S757) during IL-13–mediated mucous metaplasia and resolution 3 days after IL-13 withdrawal. (**D**–**G**) Corresponding quantification of protein levels normalized to total protein for MTOR or nonphosphorylated protein levels (S6K1, RPS6, and ULK1). *n* = 8–10 per group from *n* = 4 unique donors. Each experiment and hAEC donor is denoted with a different color. ANOVA with mixed-effect test (1-tailed) for statistical difference comparing each time point to untreated vehicle was used. *P < 0.05.

Given the complexities of MTOR signaling, we next sought to characterize the role of MTOR using an in vitro model of de novo IL-13–induced mucous cell metaplasia. IL-13 stimulation recapitulates many of the morphologic features of the diseased epithelium in severe asthma, including increased cell size, epithelial cell hyperplasia, MUC5AC staining, and formation of eGC (11, 30–35) (Supplemental Figure 1; supplemental material available online with this article; https://doi.org/10.1172/jci.insight.187904DS1). Primary human airway epithelial cells (hAEC) were fully differentiated under air-liquid interface (ALI) conditions (ALI ≥ 21 days) and examined for MTOR signaling by evaluation of multiple downstream substrates during IL-13 activation (Figure 3A). Starting at ALI day 21, hAEC were evaluated after treatment with IL-13 for +2 or +7 days or 3 days after withdrawal of IL-13 (Figure 3B). We found that MTOR protein levels themselves did not change during IL-13–mediated mucous metaplasia development and there was variability in MTOR protein levels by immunoblot across different hAEC donors (Figure 3, C and D). IL-13 increased phosphorylation of S6K1 and RPS6 at both 2 and 7 days of IL-13 days (Figure 3, C, E, and F). MTOR also negatively regulates autophagy by phosphorylation of serine residue S757 of ULK1 (20, 21). We (12, 25) and others (36, 37) previously have shown that autophagosome-related protein LC3 II levels are increased during the development of mucous cell metaplasia in hAEC treated with IL-13 in the context of lysosomal inhibition with bafilomycin A1 or chloroquine. These autophagy flux assays revealed concurrent signals of autophagy activation and inhibition of lysosome-autophagosome fusion both driving increased LC3 II levels. We hypothesized that the inhibition of autophagosome-lysosome fusion was temporally mediated by MTOR signaling (19, 38). Consistent with increased MTOR signaling during early IL-13–activation, we found a significant increase in phosphorylation of ULK1 at serine 757 in hAEC at +2 day of IL-13 treatment relative to untreated controls (Figure 3, C and G). However, during prolonged IL-13 activation and after IL-13 withdrawal, MTOR signaling is reduced and autophagy activation predominates. We next tested the role of MTOR using this time-dependent IL-13 model in the development and maintenance of airway mucous cell metaplasia associated with severe asthma.

### MTOR regulates cell proliferation, size, and the emergence of eGC during mucous metaplasia.

**Figure 4:**
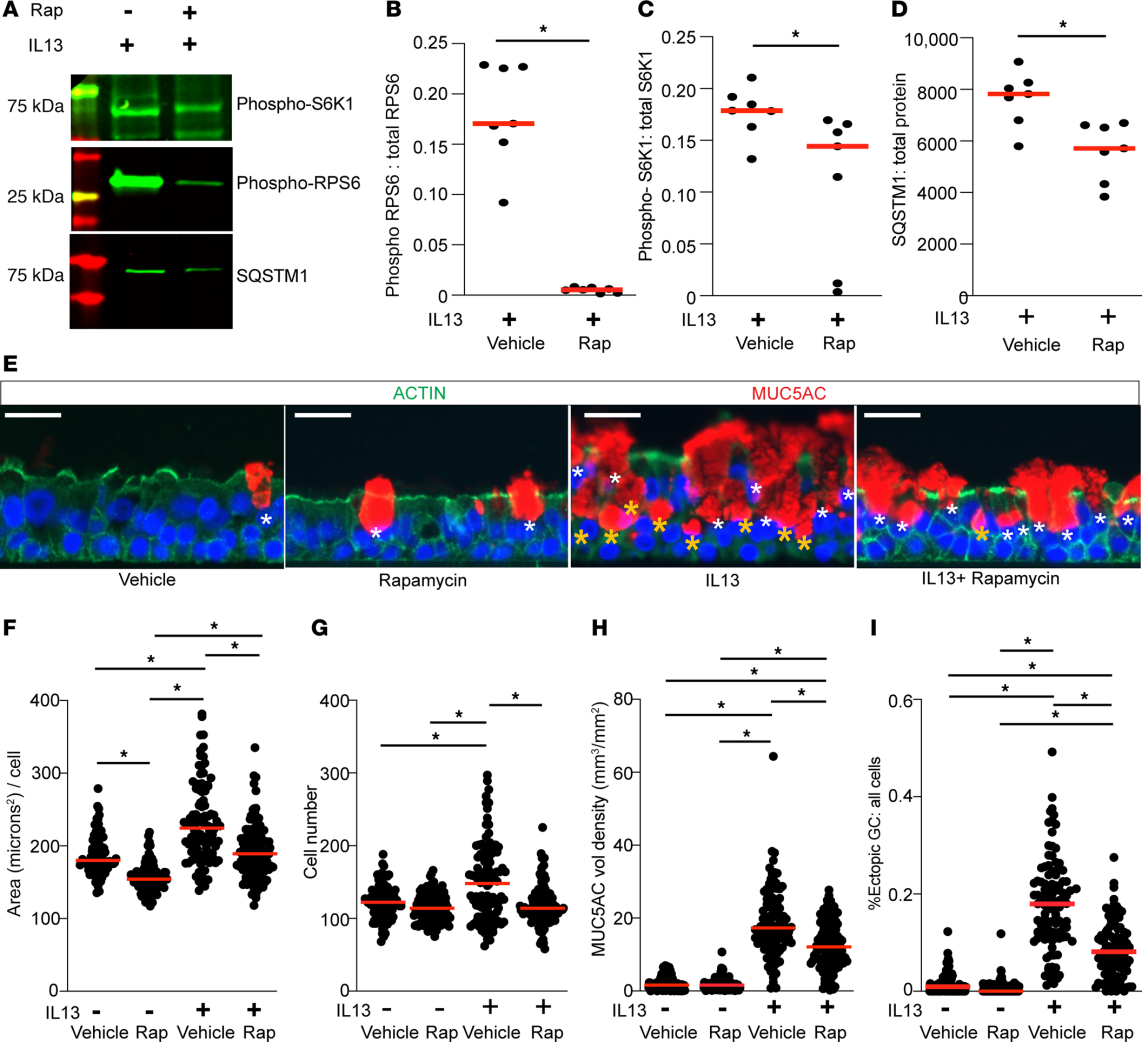
MTOR regulates airway epithelial hypertrophy, hyperplasia, and ectopic goblet cell formation. (**A**–**D**) Representative immunoblots for phosphorylated RPS6 (S-240-244), S6K1 (T389) levels, and total SQSTM1 with corresponding quantification. *n* = 7 per group from 4 hAEC donors. (**E**) Representative images of MUC5AC immunostaining for goblet cells and Actin immunostaining to define cell borders of hAEC in untreated vehicle, rapamycin alone (250 nM), IL-13 (10 ng/mL) for 7 days, and IL-13 plus concurrent rapamycin. DAPI (blue) for nuclear counterstain. Scale bars: 25 μm. White asterisk marks airway luminal surface goblet cells, golden asterisk marks airway ectopic goblet cells (eGC). (**F**–**I**) Quantification of area per cell, total number of cells, MUC5AC volume density immunostaining normalized to airway basement membrane length, eGC fraction per total cells. Images from *n* = 10–12 microscopic fields from 9 or 10 replicate inserts per condition from *n* = 6 unique normal airway donors. ANOVA (2-tailed) with Tukey multiple-comparison test for statistical difference. *P < 0.05.

To determine the functional effect of MTOR signaling in IL-13–mediated mucous cell metaplasia, we treated fully differentiated hAEC (ALI day 21) with vehicle control, MTOR inhibitor rapamycin, IL-13, or IL-13 plus rapamycin for 7 days (Supplemental Figure 2A). This concurrent model will allow examination of the role of MTOR in an IL-13–dependent and –independent fashion. When we examined morphologic changes, we found IL-13 increased airway epithelial cell size, MUC5AC immunostaining per airway, and number of eGC (Supplemental Figure 1). During IL-13 activation, concurrent rapamycin led to a significant decrease in levels of MTOR phosphorylated substrates RPS6 and S6K1, indicating that MTOR signaling was inhibited (Figure 4, A–D). In addition, rapamycin led to a decrease in levels of sequestosome-1 (SQSMT1), which is an autophagy cargo protein degraded during autophagy in the lysosome (39). This confirmed that MTOR inhibition corresponded to an increase in autophagy. Concurrent rapamycin did not change IL-13–induced signal transducer and activator of transcription 6 (STAT6) phosphorylation nor *MUC5AC* gene expression (Supplemental Figure 2, B and C). MTOR inhibition with rapamycin reduced cell size and number independently of the presence of IL-13 activation (Figure 4, E–G). However, in the presence of IL-13 activation as a model of severe asthma, MTOR signaling was required for key features of airway mucous cell metaplasia, MUC5AC staining levels, and number of eGC (Figure 4, E and G–I). There was no effect of MTOR inhibition on cilia formation either with or without IL-13 (Supplemental Figure 3). There was no corresponding increase in cleaved caspase 3 (Supplemental Figure 2D), suggesting that cellular apoptosis could not explain the rapamycin-induced decrease in cell size and number. Furthermore, the effect of MTOR inhibition on epithelial hypertrophy and hyperplasia was not due to reduction in the number of TP63^+^ basal cells (Supplemental Figure 3E). Interestingly, there was a trend toward higher TP63^+^ basal cells during concurrent IL-13 activation and MTOR inhibition. In summary, MTOR signaling was required for the development of key features of metaplastic changes including cellular hypertrophy, hyperplasia, and eGC formation.

**Figure 5:**
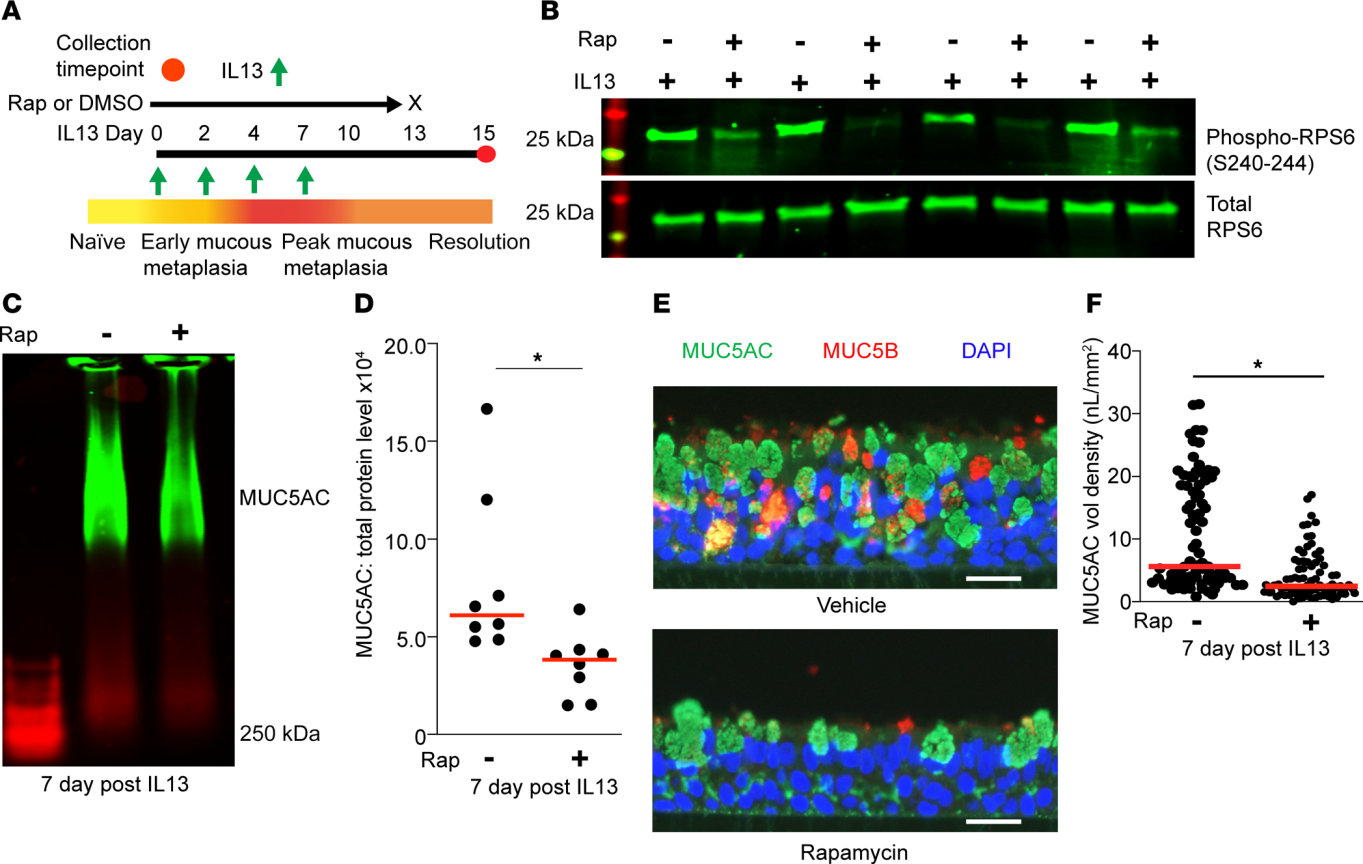
MTOR inhibition hastens resolution of IL-13–mediated mucous metaplasia by reducing MUC5AC protein levels. (**A**) Schematic for treating fully differentiated hAEC for 7 days with IL-13 (10 ng/mL) and then treated with vehicle or rapamycin (1 μM) for 48 hours following withdrawal of IL-13. (**B**) Representative immunoblots for total and phosphorylated RPS6. *n* = 4 inserts from *n* = 2 independent hAEC donors. (**C** and **D**) Representative immunoblot for MUC5AC from IL-13–treated AEC following vehicle or rapamycin treatment with corresponding quantification. *n* = 8 from 3 independent hAEC donors. (**E**) Representative immunostaining for mucin MUC5AC and MUC5B with DAPI for nuclear staining in IL-13–treated hAEC following vehicle or rapamycin treatment. Scale bar: 20 μm. (**F**) Quantification of MUC5AC volume density normalized by basement membrane length in vehicle control and rapamycin-treated hAEC. *n* = 8–10 microscopic images per hAEC donor. Six inserts from *n* = 4 unique hAEC donors. Unpaired *t* test (2-tailed) for statistical difference with *P < 0.05.

Based on our new data of short-term IL-13 stimulation leading to the development of MTOR-dependent features of mucous cell metaplasia and our prior work showing persistent increase in autophagy and ROS levels during prolonged IL-13 stimulation, including after cytokine withdrawal (12, 25), we hypothesized that epithelial cells would be more responsive to autophagy activation during resolution of mucous metaplasia. We have previously demonstrated that autophagy contributes to the resolution of mucous metaplasia through the degradation of mucin granules in the lysosome (40). We predicted that MTOR inhibition would accelerate the degradation of mucin granules. hAEC were treated with IL-13 for 7 days, and the cytokine was withdrawn to model resolution. hAEC were treated with a single high-dose rapamycin or vehicle control for 48 hours and then harvested at day 7 after cytokine withdrawal (Figure 5A). There was a reduction in phosphorylated-RPS6 by immunoblot, indicating MTOR inhibition (Figure 5B). We found that MTOR inhibition with rapamycin significantly decreased MUC5AC levels by immunoblotting and immunostaining (Figure 5, C–F). These data indicate that activating autophagy by MTOR inhibition hastens resolution and confirms that the temporal balance of MTOR and autophagy is critical in regulating key features of mucous cell metaplasia.

### MTOR regulates epithelial cell migration during IL-13–mediated mucous cell metaplasia.

**Figure 6:**
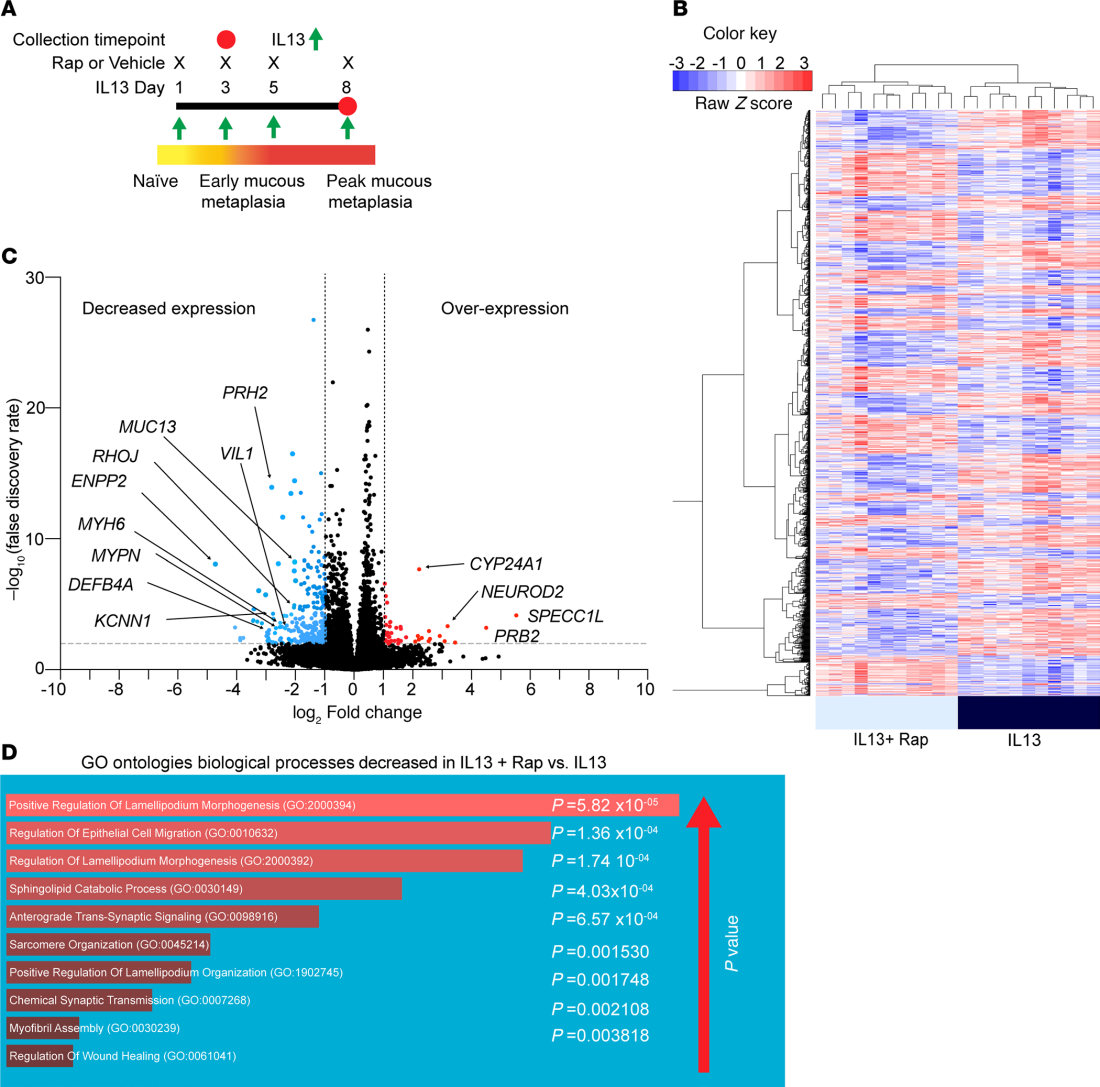
MTOR inhibition leads to decreased expression of cytoskeletal organization and migration genes during IL-13 stimulation. (**A**) hAEC under ALI conditions were treated with IL-13 in the presence or absence of concurrent rapamycin (250 nM) for 8 days. (**B**) Heatmap of hAEC from IL-13 (10 ng/mL) versus IL-13 plus rapamycin condition for differentially expressed genes (DEG) adjusted *P* < 0.05. *n* = 5 unique donors with *n* = 2–3 replicates per donor. (**C**) Volcano plot showing log_2_ fold change for DEG identified from **B**. (**D**) GO Annotation pathways analysis of 40 genes log_2_ 2-fold decreased DEG from the IL-13 plus rapamycin group versus IL-13 alone from **C**. Pathways are on the left of the figure with rank order of significance by corresponding *P* < 0.5.

To explore fundamental MTOR-dependent pathways during IL-13–mediated mucous metaplasia, we undertook RNA-Seq of IL-13–stimulated hAEC in the presence or absence of concurrent MTOR inhibition by rapamycin (Figure 6A). We found a large set of significant differentially expressed genes (DEG) between the 2 treatment groups (Figure 6B and Supplemental Data 1). A comparison among these DEG with the highest difference (≥log_2_ fold change) revealed that MTOR inhibition by rapamycin was associated with a decrease in expression of genes related to cytoskeletal organization and epithelial migration, including *VIL1*, *RHOJ*, *KCNN1*, *MUC13*, and *ENPP2*, among others (Figure 6C). Gene ontology (GO; https://geneontology.org) of biological processes confirmed that genes involved with cytoskeletal organization and cell migration were decreased in the IL-13 plus rapamycin treatment group (Figure 6D and Supplemental Data 2). To further characterize the functional role of MTOR in epithelial cell migration, we assessed epithelial migration in response to mechanical wounding assays using an undifferentiated hAEC model. MTOR inhibition with rapamycin in the presence of IL-13 significantly decreased the percentage of wound closure at both the 6- and 24-hour time points (Supplemental Figure 4, A–D). This suggested that MTOR signaling plays an important role in epithelial migration in an injury repair model.

**Figure 7:**
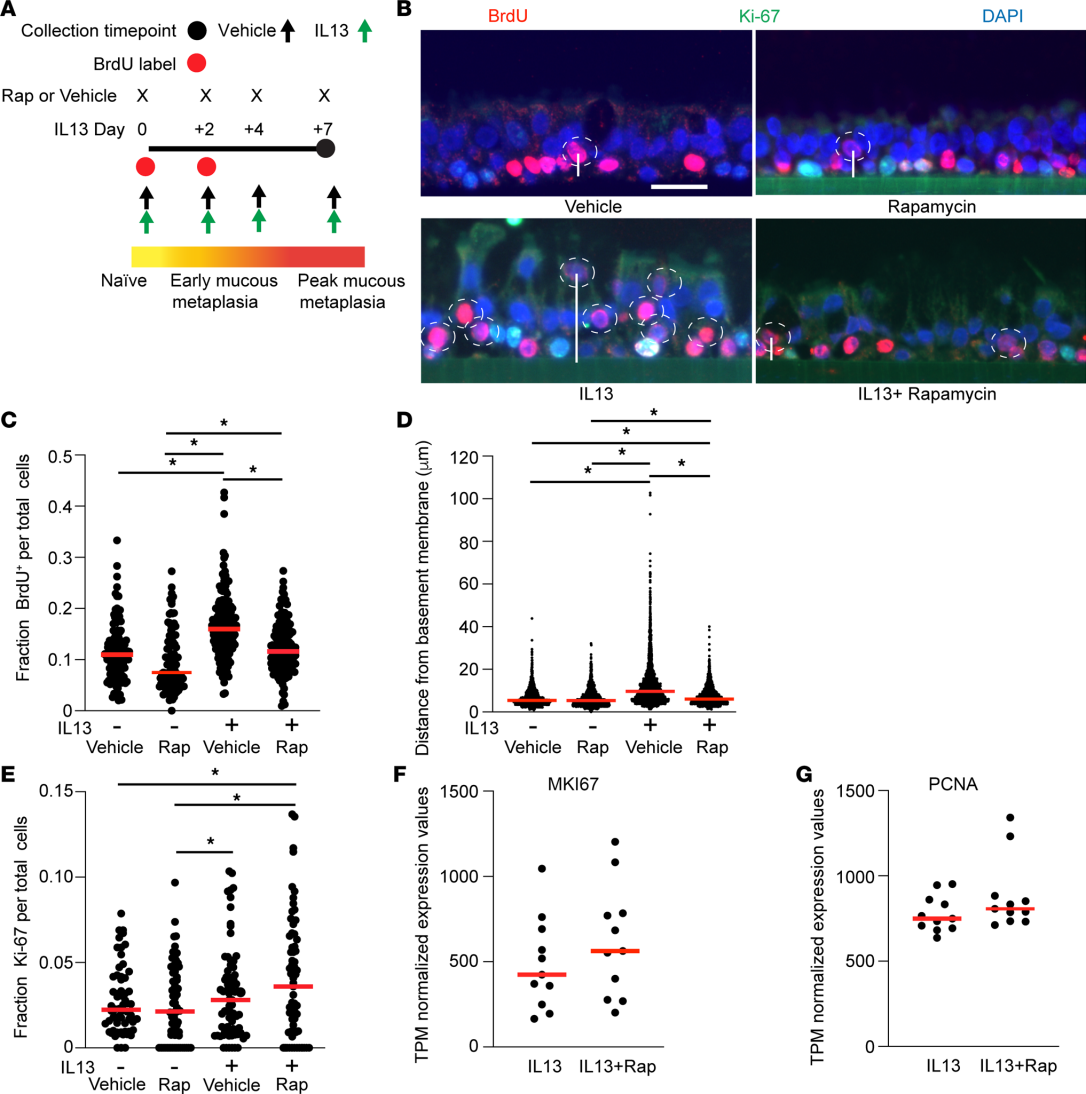
MTOR regulates movement of airway epithelial cells during IL-13–mediated mucous cell metaplasia. (**A**) Schematic showing timing of BrdU labeling and withdrawal during IL-13 mediated mucous metaplasia. (**B**) Representative Ki-67 and BrdU immunostaining with DAPI for nuclear staining in hAEC cells for 4 conditions: vehicle DSMO control, rapamycin, IL-13 for 7 days (10 ng/mL), and IL-13 plus rapamycin 7 days. Dashed circles indicate migrating BrdU labeled cells. Scale bar: 25 μm. (**C**) Quantification of fraction of BrdU-labeled cells compared with total cell number per image. (**D**) Quantification of mean distance (μm) of each BrdU-labeled cell from the basement membrane (represented by a vertical bar). (**E**) Quantification of fraction of Ki-67^+^ cells relative to total cells per image. *n* = 10 or 12 replicates from *n* = 6 unique hAEC donors with 10–15 microscopic images per airway segment. ANOVA (2-tailed) with Tukey multiple-comparison test for statistical difference. *P < 0.05. (**F** and **G**) Gene expression data for *MKI67* (**F**) and *PCNA* (**G**) was compared from the bulk RNA-Seq dataset.

Previous studies using an injury repair model have demonstrated inconsistent roles of IL-13 on epithelial wound closure (8, 9). To further explore the effect of MTOR on migration during mucous cell metaplasia independently of cellular injury in fully differentiated airway epithelial cells, we utilized BrdU labeling in hAEC under ALI conditions treated with vehicle control, rapamycin, IL-13, or concurrent IL-13 + rapamycin for 7 days (Figure 7A). BrdU was removed from the culture media after two IL-13 treatments. This approach allowed us to focus on the migration of previously labeled cells rather than ongoing proliferation at day 7 of IL-13 stimulation when the cells are harvested. Under control conditions, BrdU-labeled cells were predominately along the basement membrane. Under MTOR inhibition conditions, rapamycin led to a significant decrease in the total number of BrdU-labeled cells independent of IL-13 (Figure 7, B and C). In response to IL-13, we found a significant increase in the number of BrdU-labeled hAEC. These BrdU-labeled cells were also more apically dispersed in the parabasal epithelial layer, rather than only along the basement membrane (Figure 7, B and D). These data suggest that, under conditions of IL-13–mediated mucous cell metaplasia, there is basal-apical migration of labeled cells independent of mechanical injury. Concurrent MTOR inhibition with rapamycin significantly reduced the IL-13–driven basal-apical migration of BrdU-labeled cells. Instead, BrdU-labeled cells were primarily concentrated along the basement membrane comparable to vehicle control conditions (Figure 7, B and D). We next sought to examine the role of proliferation in this model system. There were few costained Ki-67 and BrdU labeled cells. This likely reflects the timing of BrdU withdrawal in our model system (Figure 7A). There was a nonsignificant trend toward a higher number of Ki-67^+^ cells with IL-13 activation. However, concurrent MTOR inhibition and IL-13 activation did not lead to a reduction in Ki-67 staining (Figure 7, B and E). This trend was confirmed when we examined gene expression from the same conditions in the bulk RNA-Seq data set for *MKI-67* and *PCNA* genes (Figure 7, F and G). These findings indicate that inhibiting MTOR led to a loss of migration of airway epithelial cells during IL-13–mediated mucous cell metaplasia.

### MTOR activation is necessary but not sufficient to drive mucous cell metaplasia in mouse airway cells.

**Figure 8:**
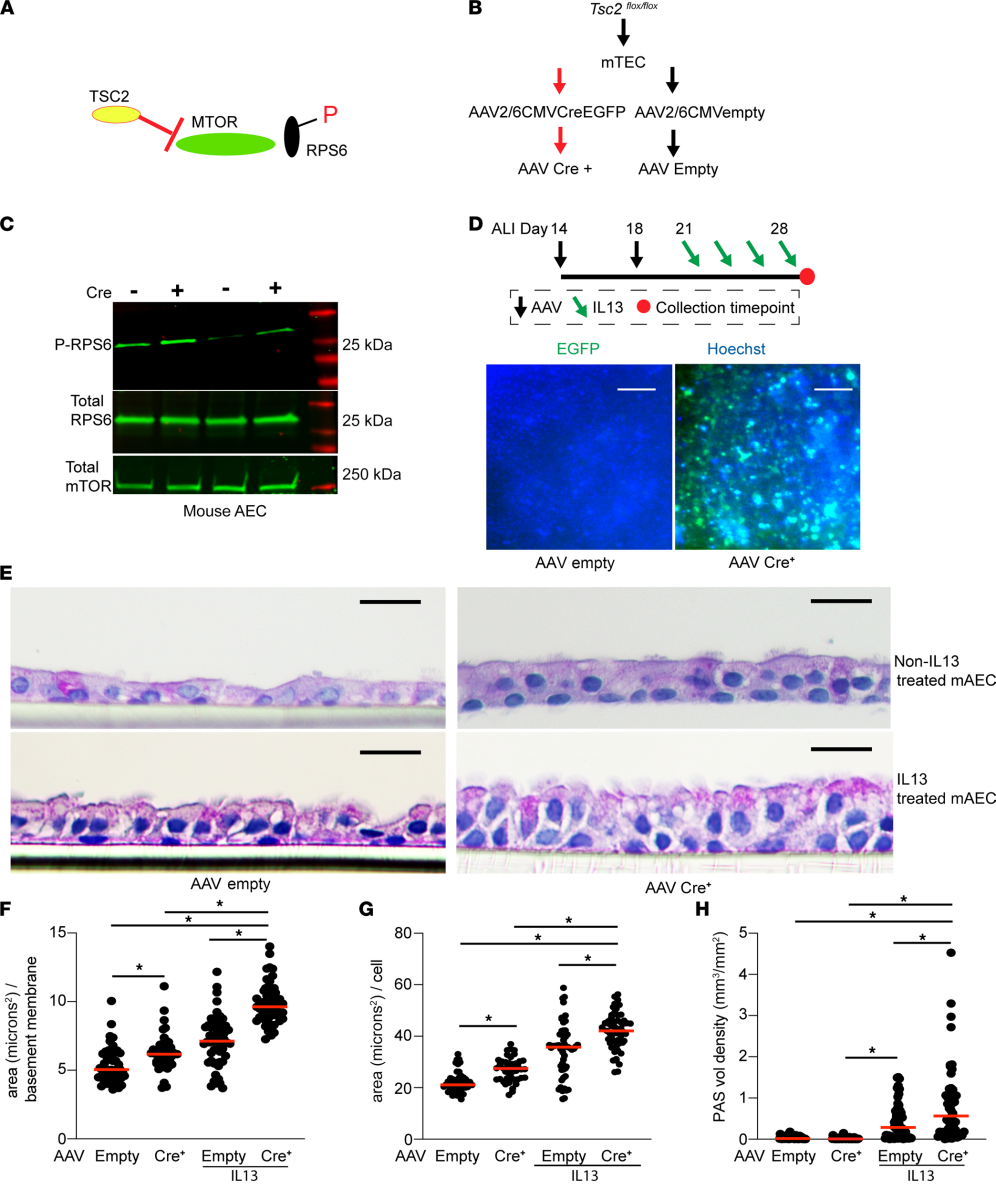
MTOR activation by Tsc2 deletion leads to increased mucous metaplasia and epithelial cell hypertrophy. (**A**) Illustration demonstrating the effect of Tsc2 deletion on MTOR activity. (**B**) Schematic for AAV transduction of mouse *Tsc2^fl/fl^* airway epithelial cells (AECs). (**C**) Immunoblotting for total and phosphorylated RibS6 and total MTOR level. *n* = 2 per group. (**D**) Schematic for repetitive transduction of AAV of *Tsc2^fl/fl^* mAEC under ALI conditions and subsequent IL-13 activation (10 ng/mL). Lower panel shows representative EGFP signal in CMV-Empty and CMV-Cre-EGFP AAV transduced cells. Scale bar: 100 μm. (**E**) Representative PAS images of AAV Empty and AAV Cre^+^ *Tsc2^fl/fl^* mAECs with and without IL-13 treatment. Scale bar: 10 μm. (**F**–**H**) Quantification of total epithelial area per airway (**F**), area per cell (**G**), and PAS staining volume density immunostaining normalized to airway basement membrane length (**H**). *n* = 10–12 images per insert, *n* = 5 inserts derived from 3 Tsc2 flox mice. ANOVA (2-tailed) with Tukey multiple-comparison test for statistical difference. *P < 0.05.

To complement the pharmacologic loss-of-function studies using rapamycin, we sought an upstream MTOR activator using a genetic model. Tuberous sclerosis complex 2 (Tsc2) encodes the MTOR-suppressing protein tuberlin (41–44). Mice with a conditional deletion of Tsc2 have increased MTOR signaling in the presence of Cre activation (45). Mouse tracheal *Tsc2 ^fl/fl^*-derived AECs were propagated in culture and found to have the expected increase in phosphorylated RPS6 levels upon AAV2-6-mediated Cre activation (Figure 8, A–C). To determine the effect on mucous cell metaplasia, *Tsc2^fl/fl^* mouse AECs that had been transduced with either AAV2-6 control CMV-Empty or AAV2-6 CMV-Cre-EGFP viral particles were treated with mouse recombinant IL-13 or vehicle control. Transduction efficiency was confirmed by EGFP signal at ALI day 28 (Figure 8D). Mouse AAVCre^+^ *Tsc2^fl/fl^* AECs had significant increases in airway cell hypertrophy and mucous cell metaplasia as measured by PAS staining (Figure 8, E–H). Comparable with our rapamycin hAEC model, the effect of MTOR activation cell size was independent of IL-13. However, MTOR activation alone was insufficient to increase mucous cell metaplasia independently of IL-13 (Figure 8, E and H). Taken together, these findings indicate the central role of MTOR signaling in perturbing key features of epithelial control of cell size, organization, and differentiation that contribute to airway obstruction in asthma.

## Discussion

In airway disease such as severe asthma, there is an increase in epithelial hyperplasia, hypertrophy, and mucous cell metaplasia, all contributing to airway wall thickening, mucous plugging, and loss of lung function (4, 5, 46). Morphologically, these alterations were observed in the airway sections from those with severe asthma. At a cellular level, these pathogenic changes are the result of a loss of control of size, morphology, migration, and differentiation. These pathogenic changes are initiated by allergens, viruses, and type 2 inflammatory cytokines such as IL-13. Changes in airway epithelial cell fate and differentiation by type 2 inflammatory conditions have been studied (30, 47). However, the maladaptive epithelial response that leads to loss of control of cell size, proliferation, and migration is not well understood. In addition, we (12, 25) and others (36, 48–50) have observed that autophagy proteins are increased in the airway epithelium from asthma or in response to IL-13. However, autophagy is predominately a catabolic process in response to cell stress, including oxidant injury. To date, it has been challenging to reconcile the morphologic and differentiation changes in severe asthma with autophagy activation. Here, we propose what we believe to be a new paradigm: that the balance of MTOR and autophagy determines the development, persistence, and resolution of airway mucous cell metaplasia found in severe asthma.

In airway sections from individuals with severe asthma, we found an increased airway epithelial thickness and mucous cell metaplasia marked by significant increases in MUC5AC staining of GC. Moreover, there were regions of epithelial hypertrophy or bulges of cell clusters with a high density of eGC. The MTOR pathway was critical to the development of these morphologic abnormalities. First, MTOR phosphorylation of substrate RPS6 was increased along the epithelium from individuals with severe asthma, particularly in regions rich in GC. Second, primary hAEC differentiated under ALI conditions and treated with IL-13 in a de novo mucous cell metaplasia model demonstrated a temporal relationship with MTOR substrate phosphorylation. Early IL-13–mediated activation increased MTOR signaling and inhibited autophagy signaling through ULK1. However, after the withdrawal of IL-13, the MTOR substrate signaling declined. This corresponded to our previous data showing persistent autophagy activation and ROS during prolonged IL-13 stimulation, including even after cytokine withdrawal during resolution. Manipulating the balance of MTOR and autophagy may be a therapeutic pathway to pursue in severe asthma. Treatment of hAEC with a single dose of rapamycin following IL-13–mediated mucous cell metaplasia reduced MUC5AC levels and accelerated resolution. This finding confirmed our previous work showing that MTOR inhibition reduced MUC5AC levels in COPD cells (40). Fourth, using rapamycin to inhibit MTOR signaling in human AEC or genetic deletion of Tsc2 in mouse AECs, we identified that airway epithelial proliferation, cell size control, and mucous cell metaplasia were dependent on MTOR signaling. Control of cell size and proliferation was independent of IL-13 stimulation. However, critical features of airway mucous cell metaplasia, including MUC5AC levels and a morphologically distinct population of eGC, were dependent on IL-13 signaling upstream of MTOR. Finally, using 2 distinct migration assays either dependent or independent on mechanical injury, we found that MTOR regulated epithelial cell migration. Traditional epithelial injury repair models have led to conflicting reports of the role of IL-13 on migration, depending on the state of differentiation. Previously, it has been shown that IL-13 induces increased cellular migration (9) in wound assays for undifferentiated hAEC. More recent data in well-differentiated AEC indicate that IL-13 may negatively regulate migration in an injury repair model (8). To understand the role of IL-13 on airway epithelial cell migration independent of physical injury and repair, we used BrdU in our IL-13 hAEC model with early withdrawal of BrdU from the media to focus on migration of labeled cells. BrdU labeling demonstrated the importance of cell basal-apical migration in the context of IL-13–mediated mucous cell metaplasia, independent of cell injury which is relevant to type 2 asthma pathogenesis. The association of MTOR-dependent morphological changes with cellular migration and cytoskeletal organization was supported by bulk RNA-Seq showing decreased expression of genes *VIL1*, *RhoJ*, *ENPP2*, *MUC13*, and *KCNN1*, among others, involved with cytoskeletal organization and migration. The association with MTOR signaling and airway epithelial cell migration in the context of type 2 inflammation was an unexpected and potentially novel finding of our study. Previous work has identified a role for MTOR in the lung regulating lung branching morphogenesis during normal development (51) and in disease pathogenesis by mesenchymal cell migration in lymphangioleiomyomatosis (LAM) (52). Recent data suggest that MTORC2 also has a role in regulating cellular orientation and migration; however, we did not examine that signaling pathway (53–55). Future studies are warranted to dissect the unique roles of MTORC1 and MTORC2 in each cell type using mouse genetic models and single-cell RNA-Seq (scRNA-Seq) of hAEC under conditions of MTOR inhibition.

Recent work with single-cell transcriptomics has led to the identification and characterization of subpopulations of airway epithelial cells with distinct roles, including distinct populations of GC (1). During the recurrent airway inflammation associated with asthma, the normal differentiation paradigm is perturbed, leading to altered differentiation program as well as distinct GC populations (2). The transcriptional changes by type 2 stimulation (2), and specifically IL-13 (30, 47), have also been characterized. These data point to broad epithelial–type 2 inflammatory cell interactions and paracrine growth factor signaling between epithelial cells. MTOR signaling may play that important paracrine signaling role within and between cells. However, existing bulk and scRNA-Seq datasets have not evaluated the potential role of intrinsic epithelial paracrine factors that lead to mucous cell metaplasia. MTOR signaling is known to play a key role in proliferation, cell growth, and protein synthesis in response to metabolic and inflammatory cues (15, 56). Severe asthma is associated with changes to the airway epithelium, submucosal gland hypertrophy, increased laminal propria, and airway fibrosis. These changes are collectively referred to as airway remodeling. Here we identified a significant increase in MTOR signaling in the airway epithelium. While not a focus of this study, it may be that MTOR signaling drives all of the factors that contribute to airway remodeling (57, 58). Further studies are needed to assess the balance of MTOR and autophagy on all phases of airway remodeling.

The first report on the role of MTOR in asthma mouse models was 20 years ago. It was observed that systemic administration of MTOR inhibitor, SAR943, significantly reduced OVA-mediated airway inflammation, epithelial proliferation, and the number of GC (59). Other studies have confirmed the importance of MTOR in type 2 models of asthma in mice (60, 61). The role of MTOR signaling in increasing airway hyperplasia was further described in phosphatase and tensin homolog (PTEN) deletion mice (62, 63). PTEN negatively regulates MTOR activity, and conditional deletion of PTEN in SPC^+^ cells led to increased proliferation in the distal airway epithelium. In contrast, a recent study (64) reports a different finding with decreased phospho-RPS6 in asthma airway sections, mouse lungs, and undifferentiated hAEC. However, there are several distinctions worth noting. First, in that study, hAEC were undifferentiated at the time of IL-13 stimulation. Second, the authors used an antibody to a different phosphorylation site phospho-RBS6 (threonine 236), which may account for the divergent findings. Earlier reports indicate that RPS6 could be phosphorylated at T235 and T236 independently of MTOR-mediated P70S6K signaling (65). Our study confirmed MTOR activation with multiple different downstream phosphorylation targets, including P70S6K, RPS6, and ULK1. Lastly, the morphologic changes found in airway epithelial cell mucous metaplasia — cell proliferation, hypertrophy, and protein synthesis — are typically associated with a high MTOR signaling state and supported by our use of both gain- and loss- of function models.

Our study has several limitations worth noting. We utilized hAEC and nonasthma control airway sections from participants whose lungs were rejected for transplantation and may have been subject to unknown environmental exposures or prolonged ventilator support time that may affect airway epithelial differentiation and organization. Second, while the IL-13 model for type 2 asthma is well accepted and reproducible, it does not capture the complex inflammatory milieu typical of the asthmatic airway, nor do our findings address nonatopic asthma. Third, we utilize the pharmacologic loss-of-function studies with rapamycin. The consequences of off-target effects from rapamycin appear to be minimal. A recent study comparing genomic and proteomic differences in cells containing normal or MTOR mutant protein showed rapamycin did not affect nonfunctional MTOR (66). This finding supports the specificity rapamycin action is specific only to the MTOR pathway. Lastly, it is not known if MTOR-dependent migration is cell specific in differentiating emergent eGC or occurs broadly across multiple cell lineages. Further studies using scRNA-Seq approaches or cell-specific lineage tracing mouse models are needed to answer this question.

In conclusion, asthma is a multifaceted condition marked by recurrent and progressively persistent airway obstruction, driven by inflammation and infection affecting airway epithelial cells, stromal cells, and surrounding inflammatory cells. Here, we propose a new paradigm centered on the importance of MTOR signaling in the development of multiple and eclectic airway epithelial phenotypes characteristic of type 2 asthma. This model was able to reconcile with our earlier work, demonstrating the importance of chronic and persistent IL-13 signaling on oxidant stress and autophagy activation. Ultimately, control of the balance of MTOR and autophagy may offer a new therapeutic strategy to treat muco-obstructive lung diseases such as asthma.

## Methods

### Sex as a biological variable.

Our study examined male and female animals, and similar findings are reported for both sexes. Similar numbers of lungs from male and female donors were used for immunostaining in Figures 1 and 2 (Table 1). Eight unique human AEC donors (5 males and 3 females) were used to collect primary hAEC for staining, bulk RNA-Seq, AEC migration, and immunoblotting studies. For mouse *Tsc2^fl/fl^* airway epithelial cells used in migration and ALI culture, tracheas from 3 males and 2 females were mixed in a common pool and expanded as previously described (57, 58).

### HAECs.

Human AEC cultures were derived from airways of donor lungs not suitable for transplant, seeded at 1.25 × 10^5^ cells per 454.4 mm^2^ surface area, and differentiated on membrane-supported Corning Transwell (Millipore Sigma, CLS3401-48EA) inserts under ALI conditions as previously described (12, 25, 67). BEGM growth media (Lonza, CC 3171) was used for proliferation until cells were confluent, and then media were transitioned to PneumoCult ALI media (Stemcell Technologies, 05001). Upon full differentiation (ALI days 21–28), hAEC were treated with human recombinant IL-13 (10 ng/mL) (BioLegend, 571102) and/or rapamycin (250 nM, 1 μM) (MilliporeSigma, 553210) for the indicated times.

### Approval of clinical samples.

Lung tissues were obtained from individuals who had a history of asthma and died of fatal asthma attacks. These lungs were not considered usable for lung transplantation and were provided through materials transfer agreements from the International Institute for the Advancement of Medicine (IIAM) or from Mid-America Transplant (St. Louis, Missouri, USA) organ procurement organization. Lungs were deidentified, and consent for research was obtained from next of kin according to local organ procurement organization standards. Sections were provided courtesy of Derek Byers (Washington University School of Medicine). Characteristics are provided in Table 1.

Human AECs derived from healthy lungs not suitable for transplant were isolated under protocols approved by the University of Nebraska Medical Center (UNMC). Regarding the sources of tissue: UNMC Division of Pulmonary, Critical Care, and Sleep Medicine maintains a tissue bank that stores lung tissue, airway histological sections, bronchoalveolar lavage fluid (BALF), and blood samples from deidentified nondiseased lungs and trachea not suitable for transplant accepted from the International Institute for the Advancement of Medicine (IIAM), the National Disease Research Interchange (NDRI), and Live On Nebraska. All 3 nonprofit groups provide deidentified tissue for research. No identifiers are provided, and identifiers are not accessible from the repository. This protocol is approved by the UNMC IRB, protocol #0077-25-EP.

### Mouse Tsc2^fl/fl^ AECs.

All mice experiments were performed according to corresponding guidelines and regulations including ARRIVE guidelines (68, 69). Mouse airway epithelial cells (mAECs) were derived from Tubular Sclerosis Complex 2 (*Tsc2*) floxed mouse tracheas. Mice originally from The Jackson Laboratory (*Tsc2^tm1.1Mjg^/J* strain no. 027458) and were provided by Kory Lavine from Washington University School of Medicine. *Tsc2^fl/fl^* tracheas were excised and mouse AECs were collected according to previously published protocols (40). mAECs were digested and propagated in media + Rho kinase inhibitor Y27632 (Tocris, TB1254-GMP) and keratinocyte growth factor (KGF) (BioLegend, 752204) on 6 mm Transwell inserts for differentiation under ALI conditions as previously described (40). After 14 days of ALI conditions, *Tsc2*^fl/fl^ AECs were transduced either with AAV 6/2 CMV;Cre;EGFP or AAV6/2 Empty virus control (Iowa Vector Core nos. 2490 and 7483). After 5 days of culture, a second transduction of AAV was performed, and mouse recombinant IL-13 (10 ngm/mL) (BioLegend 571102) was added to the culture media. Transduction efficiency was measured either by EGFP signal using live cell immunofluorescent imaging or changes to MTOR immunoblotting for phospho RPS6 as described below.

### Immunoblot analysis.

MTOR substrate proteins were collected from AECs. NP-40 buffer supplemented with protease/phosphatase inhibitors was used for lysis to preserve phosphorylation sites. The following antibodies were used for detection of protein on PVDF membranes following transfer: total MTOR (Cell Signaling Technology, 2972S), P70S6K total and T389 phosphorylation (Cell Signaling Technology, 9202S and 9205S), RPS6 total and S240, 244 phosphorylation (Cell Signaling Technology, 2217S and 2215S), ULK1 total and S757 phosphorylation (Cell Signaling Technology, 8054S and 14202S), and STAT6 total and Y641 phosphorylated (Cell Signaling Technology, 9362S and 9361S). Signal was quantified with infrared (IR) labeled secondary antibodies (LiCor) and normalized to total protein. In order to multiplex 4 different MTOR substrate proteins from the same Western blot, the membrane was cut prior to the blocking step based on molecular weight markers. To detect mucin MUC5AC, by immunoblotting, cell lysates were lysed in the cocktail of phosphate-buffered saline (PBS) plus protease inhibitors and then sonicated for 5 seconds until lysate was clear. Lysates were then centrifuged at 15,000*g* for 30 minutes. The supernatant was collected, quantified, and heat denatured prior to electrophoresis on 0.8% agarose gel for 90 minutes at 115 mV. Protein lysates were transferred by vacuum to nitrocellulose membrane for 2 hours at 8–10 psi. After blocking in a solution of 5% milk in PBS, membranes were probed with mouse MUC5AC -45M1 (MilliporeSigma, MS-145-P1) antibody overnight at 4°C on a rocker. After PBS washes, the membranes were probed with goat anti–mouse IR conjugated antibody (LiCor, 926-32213; LiCor, 926-32210), and bands were normalized to total protein stain for quantification.

### IHC.

Slides sectioned from formalin-fixed and paraffin-embedded tissue. They were incubated in 3 washes of xylene followed by 3 washes of isopropanol. Slides were then washed in deionized H_2_O, followed by antigen retrieval with Trilogy (MilliporeSigma, 922P-06). Slides were then rinsed in tris-buffered saline plus 0.1% triton (TBST), followed by endogenous peroxidases quenching with 3% H_2_O_2_. Slides were then rinsed in tris buffered saline with 0.5% triton (TBST) and blocked with 10% normal goat serum (Vector, S-1000) in phosphate-buffered saline (PBS) for 30 minutes at 37°C in a humidity chamber. Slides were then incubated with primary antibody Phospho-RPS6 (Cell Signaling Technology, 2215S) diluted in 0.5% bovine serum albumin (BSA) in PBS overnight at 4°C. Slides were then rinsed in TBST, followed by biotinylated goat secondary antibody (Vector, BA-1000) in the same blocking buffer. After washing the slides in PBS, ABC reagent (Vector PK-7100) was used, followed by chromogen substrate staining-DAB (Vector SK-4105). Slides were washed in PBS and counterstained with hematoxylin, washed in water, and dehydrated with increasing concentrations of ethanol incubations and then xylene. To subclassify asthma airway sections into regions of GC-low and GC-rich areas, we used morphologic classification of GC along with a large apical translucent cytoplasm consistent with stored mucin granules. GC-rich regions were classified as estimated > 50% GC and GC-low regions as < 50% estimated GC.

### Immunofluorescence staining.

Human AECs on Corning Transwell membrane supports were harvested at the indicated time points. Basal media was removed, and 200 μL of 37°C–40°C 1% agarose was added to the apical surface. After cooling, the cells on membrane supports were fixed in 10% formalin and embedded in paraffin. AEC inserts and human airway sections were processed for immunostaining using the same method as previously described (40). Antibodies for immunostaining included: mouse anti–MUC5AC 45M1 (MilliporeSigma, MS-145-P1), rabbit anti-Actin (Cell Signaling Technology, 4970S), rabbit anti–E-Cadherin (Cell Signaling Technology, 3195S), rabbit anti–cleaved caspase 3 (Cell Signaling Technology, 9661S), rabbit anti-TP63 (Fitzgerald, 70R-12666), phospho-RPS6 (S240, 244) (Cell Signaling Technology, 2215S), and rabbit anti-BrdU (Invitrogen, PA5-32256). Image analysis was performed using set a common threshold with ImageJ as previously described (25, 40, 67). eGC were defined as MUC5AC^+^ cells from human airway or hAEC sections that were not localized along the apical surface of the airway lumen. A blinded observer made the determination of eGC and normal GC. Values were normalized by total GC and airway segment length. Normal GC were classified as being in direct contact with the luminal surface, while eGC were classified if they had no direct contact with the luminal surface.

### Bulk RNA-Seq construct preparation and analysis from hAEC.

hAEC from 5 unique human donors in duplicate were cultured with IL-13 (10 ng/mL) and/or rapamycin 250 ng/mL (MilliporeSigma, 553210) for 7 days. Cells were briefly rinsed with warm sterile PBS and 5 mM DTT. Total RNA was isolated by Qiagen Midi Kit (Qiagen, 75144) with 500–2000 ng for library preparation. RNA quality was verified 260/280 optical density ratio. Potential RNA degradation was measured by Advanced Analytical Technical Instrument Fragment Analyzer (AATI). Samples with RQN from the Fragment Analyzer that is above 8.0 were used. Sequencing libraries were generated in the UNMC Genomic Core by NuGen Universal mRNA Kit and sequenced on Novaseq 6000 sequencer. Size of insert of the resulting libraries was assessed by Bioanalyzer (Agilent Technologies). Each library had a unique indexing identifier barcode for individual libraries to be multiplexed together for sequencing. Multiplexed libraries were sequenced on a SP100 flow cell (Illumina) using a single-read protocol to generate a total of approximately 20 million reads for each sample. DEG were identified with a FDR (adjusted *P* value) < 0.05 and had ≥ log_2_ fold change (Supplemental Data 1). To identify key pathways involved with MTOR inhibition, 40 significantly decreased genes from the Rapamycin+IL-13 group versus IL-13 group were uploaded to GO Ontologies Biological Process 2023 using Enrichr (70) and (Supplemental Data 2).

### Cell migration assay.

Human AECs were expanded from passage 0 using BGEM media (Lonza). At passage 1, a total of 75,000 cells was seeded on a 24-well plate. When confluent, the 5% FBS was added to BGEM media ± IL-13 (10 ng/mL) (BioLegend 571102) and rapamycin 250 nM (MilliporeSigma, 553210). Standard circular wounds using a sterile tool were made in the center of the cell layer as previously described (71, 72). Wound closure was assessed by light field microscopy at 6- and 24-hour time points and complete closure. Wound area at each time point was normalized to the wound area at time 0.

### BrdU labeling of human AEC.

hAEC were seeded on membrane support inserts as previously described (12). When fully differentiated at approximately 21 days after ALI, hAEC were treated with ± IL-13 (10 ng/mL) and rapamycin (250 nM) (MilliporeSigma, 553210). All hAEC were treated with BrdU at a final concentration of (10 μM). A repeat BrdU treatment was then done at 48 hours. BrdU-labeled media were removed 96 hours after treatment. hAEC were harvested at day 8 after BrdU for immunostaining.

### Statistics.

Statistical analysis was performed using Prism 10.2. Methods for each analysis were described in the figure legend. *P* < 0.05 was considered statistically significant. Unpaired *t* test (2-tailed) was performed for parametric analyses. ANOVA was performed to address multiple comparisons by Tukey for parametric measures across all groups in the experiment (2-way). ANOVA with mixed-effect test for statistical difference only with the vehicle condition (1-way) for Figure 3 was performed to identify and compare each time point to untreated control.

### Study approval.

All animal studies cited herein have been approved by UNMC IACUC.

### Data availability.

Excel files with raw RNA-Seq data are uploaded as supplemental files. Values for all data points in graphs are reported in the Supporting Data Values file. Bulk RNA-Seq data comparing hAEC treated with IL-13 or IL-13 plus rapamycin (Supplemental Data File 1) has been deposited in a public repository and is accessible upon request. The accession number (DOI) is 10.5281/zenodo.15190704.

## Author contributions

KMK, LFV, SKM, and EME, designed and conducted experiments. SLB assisted with experimental design and manuscript editing. AH assisted with RNA-Seq data analysis. TAW assisted with cell migration experiments and manuscript editing. KLB assisted with primary cell culture and manuscript editing. JDD designed experiments, conducted experiments, and wrote and edited the manuscript.

## Supplementary Material

Supplemental data set 1

Supplemental data set 2

Supplemental figures 1-4

Unedited blot and gel images

Supporting data values

## Figures and Tables

**Table 1:**
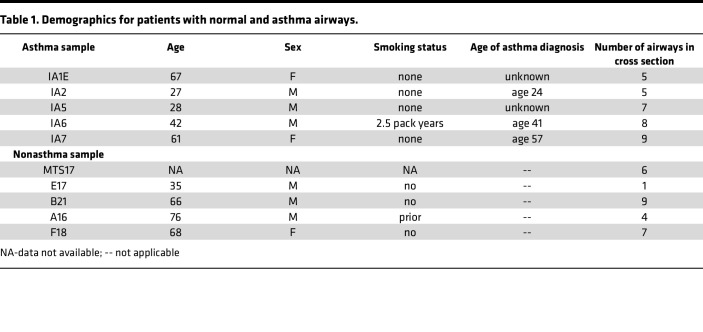
Demographics for patients with normal and asthma airways.

## Version 1 05/30/2025

In-Press Preview

## Version 2 07/08/2025

Electronic publication

## References

[1] Montoro DT (2018). A revised airway epithelial hierarchy includes CFTR-expressing ionocytes. Nature.

[2] Vieira Braga FA (2019). A cellular census of human lungs identifies novel cell states in health and in asthma. Nat Med.

[3] Deprez M (2020). A single-cell atlas of the human healthy airways. Am J Respir Crit Care Med.

[4] Aikawa T (1992). Marked goblet cell hyperplasia with mucus accumulation in the airways of patients who died of severe acute asthma attack. Chest.

[5] Ordonez CL (2001). Mild and moderate asthma is associated with airway goblet cell hyperplasia and abnormalities in mucin gene expression. Am J Respir Crit Care Med.

[6] Cohen L (2007). Epithelial cell proliferation contributes to airway remodeling in severe asthma. Am J Respir Crit Care Med.

[7] Tran C (2022). Luminal mucus plugs are spatially associated with airway wall thickening in severe COPD and asthma: a single-centered, retrospective, observational study. Respir Med.

[8] Jin M (2022). Real-time imaging of asthmatic epithelial cells identifies migratory deficiencies under type-2 conditions. J Allergy Clin Immunol.

[9] Allahverdian S (2008). Secretion of IL-13 by airway epithelial cells enhances epithelial repair via HB-EGF. Am J Respir Cell Mol Biol.

[10] Yang SJ (2019). IL-13 signaling through IL-13 receptor α2 mediates airway epithelial wound repair. FASEB J.

[11] Alevy YG (2012). IL13-induced airway mucus production is attenuated by MAPK13 inhibition. J Clin Invest.

[12] Dickinson JD (2016). IL13 activates autophagy to regulate secretion in airway epithelial cells. Autophagy.

[13] Noda T, Ohsumi Y (1998). Tor, a phosphatidylinositol kinase homologue, controls autophagy in yeast. J Biol Chem.

[14] Zoncu R (2011). mTOR: from growth signal integration to cancer, diabetes and ageing. Nat Rev Mol Cell Biol.

[15] Kim DH (2002). mTOR interacts with raptor to form a nutrient-sensitive complex that signals to the cell growth machinery. Cell.

[16] Foster KG (2010). Regulation of mTOR complex 1 (mTORC1) by raptor Ser863 and multisite phosphorylation. J Biol Chem.

[17] Wu X (2022). Beyond controlling cell size: functional analyses of S6K in tumorigenesis. Cell Death Dis.

[18] Kim J (2011). AMPK and mTOR regulate autophagy through direct phosphorylation of Ulk1. Nat Cell Biol.

[19] Jung CH (2009). ULK-Atg13-FIP200 complexes mediate mTOR signaling to the autophagy machinery. Mol Biol Cell.

[20] Hosokawa N (2009). Nutrient-dependent mTORC1 association with the ULK1-Atg13-FIP200 complex required for autophagy. Mol Biol Cell.

[21] Jung CH (2011). ULK1 inhibits the kinase activity of mTORC1 and cell proliferation. Autophagy.

[22] Klionsky DJ (2016). Guidelines for the use and interpretation of assays for monitoring autophagy (3rd edition). Autophagy.

[23] Haspel J (2011). Characterization of macroautophagic flux in vivo using a leupeptin-based assay. Autophagy.

[24] Levine B, Klionsky DJ (2004). Development by self-digestion: molecular mechanisms and biological functions of autophagy. Dev Cell.

[25] Dickinson JD (2018). Autophagy regulates DUOX1 localization and superoxide production in airway epithelial cells during chronic IL-13 stimulation. Redox Biol.

[26] Lam HC (2013). Histone deacetylase 6-mediated selective autophagy regulates COPD-associated cilia dysfunction. J Clin Invest.

[27] James AL, Wenzel S (2007). Clinical relevance of airway remodelling in airway diseases. Eur Respir J.

[28] Awadh N (1998). Airway wall thickness in patients with near fatal asthma and control groups: assessment with high resolution computed tomographic scanning. Thorax.

[29] Ruvinsky I (2005). Ribosomal protein S6 phosphorylation is a determinant of cell size and glucose homeostasis. Genes Dev.

[30] Jackson ND (2020). Single-cell and population transcriptomics reveal pan-epithelial remodeling in type 2-high asthma. Cell Rep.

[31] Wills-Karp M (1998). Interleukin-13: central mediator of allergic asthma. Science.

[32] Lee JH (2001). Interleukin-13 induces dramatically different transcriptional programs in three human airway cell types. Am J Respir Cell Mol Biol.

[33] Kim S (2002). IL-13–induced Clara cell secretory protein expression in airway epithelium: role of EGFR signaling pathway. Am J Physiol Lung Cell Mol Physiol.

[34] Kondo M (2002). Interleukin-13 induces goblet cell differentiation in primary cell culture from guinea pig tracheal epithelium. Am J Respir Cell Mol Biol.

[35] Humbert M (1997). Elevated expression of messenger ribonucleic acid encoding IL-13 in the bronchial mucosa of atopic and nonatopic subjects with asthma. J Allergy Clin Immunol.

[36] Liu JN (2016). The role of autophagy in allergic inflammation: a new target for severe asthma. Exp Mol Med.

[37] McAlinden KD (2018). Autophagy activation in asthma airways remodeling. Am J Respir Cell Mol Biol.

[38] Egan D (2011). The autophagy initiating kinase ULK1 is regulated via opposing phosphorylation by AMPK and mTOR. Autophagy.

[39] Turco E (2021). Reconstitution defines the roles of p62, NBR1 and TAX1BP1 in ubiquitin condensate formation and autophagy initiation. Nat Commun.

[40] Sweeter JM (2021). Autophagy of mucin granules contributes to resolution of airway mucous metaplasia. Sci Rep.

[41] Cai SL (2006). Activity of TSC2 is inhibited by AKT-mediated phosphorylation and membrane partitioning. J Cell Biol.

[42] Huang J (2008). The TSC1-TSC2 complex is required for proper activation of mTOR complex 2. Mol Cell Biol.

[43] Inoki K (2002). TSC2 is phosphorylated and inhibited by Akt and suppresses mTOR signalling. Nat Cell Biol.

[44] Rachdi L (2008). Disruption of Tsc2 in pancreatic beta cells induces beta cell mass expansion and improved glucose tolerance in a TORC1-dependent manner. Proc Natl Acad Sci U S A.

[45] Hernandez O (2007). Generation of a conditional disruption of the Tsc2 gene. Genesis.

[46] Fahy JV (2001). Remodeling of the airway epithelium in asthma. Am J Respir Crit Care Med.

[47] Koh KD (2023). Genomic characterization and therapeutic utilization of IL-13–responsive sequences in asthma. Cell Genom.

[48] Zhao J (2020). PEBP1 acts as a rheostat between prosurvival autophagy and ferroptotic death in asthmatic epithelial cells. Proc Natl Acad Sci U S A.

[49] Nagasaki T (2022). 15LO1 dictates glutathione redox changes in asthmatic airway epithelium to worsen type 2 inflammation. J Clin Invest.

[50] Inoue D (2011). Inducible disruption of autophagy in the lung causes airway hyper-responsiveness. Biochem Biophys Res Commun.

[51] Zhang K (2022). mTORC1 signaling facilitates differential stem cell differentiation to shape the developing murine lung and is associated with mitochondrial capacity. Nat Commun.

[52] Lin SM (2023). Hyperactive mTORC1 in lung mesenchyme induces endothelial cell dysfunction and pulmonary vascular remodeling. J Clin Invest.

[53] Saha S (2023). Mechanosensitive mTORC2 independently coordinates leading and trailing edge polarity programs during neutrophil migration. Mol Biol Cell.

[54] Gulhati P (2011). mTORC1 and mTORC2 regulate EMT, motility, and metastasis of colorectal cancer via RhoA and Rac1 signaling pathways. Cancer Res.

[55] Goncharova EA (2014). Tumor suppressors TSC1 and TSC2 differentially modulate actin cytoskeleton and motility of mouse embryonic fibroblasts. PLoS One.

[56] Chiang GG, Abraham RT (2005). Phosphorylation of mammalian target of rapamycin (mTOR) at Ser-2448 is mediated by p70S6 kinase. J Biol Chem.

[57] Mason EC (2023). Activation of mTOR signaling in adult lung microvascular progenitor cells accelerates lung aging. J Clin Invest.

[58] Houssaini A (2018). mTOR pathway activation drives lung cell senescence and emphysema. JCI Insight.

[59] Fujitani Y, Trifilieff A (2003). In vivo and in vitro effects of SAR 943, a rapamycin analogue, on airway inflammation and remodeling. Am J Respir Crit Care Med.

[60] Mushaben EM (2011). Rapamycin attenuates airway hyperreactivity, goblet cells, and IgE in experimental allergic asthma. J Immunol.

[61] Zhang Y (2017). Activation of the mTOR signaling pathway is required for asthma onset. Sci Rep.

[62] Dave V (2008). Conditional deletion of Pten causes bronchiolar hyperplasia. Am J Respir Cell Mol Biol.

[63] Flodby P (2017). Region-specific role for Pten in maintenance of epithelial phenotype and integrity. Am J Physiol Lung Cell Mol Physiol.

[64] Li W (2020). MTOR suppresses autophagy-mediated production of IL25 in allergic airway inflammation. Thorax.

[65] Pende M (2004). S6K1(–/–)/S6K2(–/–) mice exhibit perinatal lethality and rapamycin-sensitive 5′-terminal oligopyrimidine mRNA translation and reveal a mitogen-activated protein kinase-dependent S6 kinase pathway. Mol Cell Biol.

[66] Artoni F (2023). Unbiased evaluation of rapamycin’s specificity as an mTOR inhibitor. Aging Cell.

[67] Dickinson JD (2019). MyD88 controls airway epithelial Muc5ac expression during TLR activation conditions from agricultural organic dust exposure. Am J Physiol Lung Cell Mol Physiol.

[68] Kilkenny C (2010). Improving bioscience research reporting: the ARRIVE guidelines for reporting animal research. PLoS Biol.

[69] Percie du Sert N (2020). The ARRIVE guidelines 2.0: updated guidelines for reporting animal research. PLoS Biol.

[70] Chen EY (2013). Enrichr: interactive and collaborative HTML5 gene list enrichment analysis tool. BMC Bioinformatics.

[71] Spurzem JR (2002). Activation of protein kinase A accelerates bovine bronchial epithelial cell migration. Am J Physiol Lung Cell Mol Physiol.

[72] Spurzem JR (2005). Ethanol treatment reduces bovine bronchial epithelial cell migration. Alcohol Clin Exp Res.

