# Supplementary figures and images for: MTOR signaling regulates the development of airway mucous cell metaplasia associated with severe asthma
Source: JCI Insight. 2025 May 29;10(13):e187904. doi: 10.1172/jci.insight.187904 (PMC12288895; doi:10.1172/jci.insight.187904)

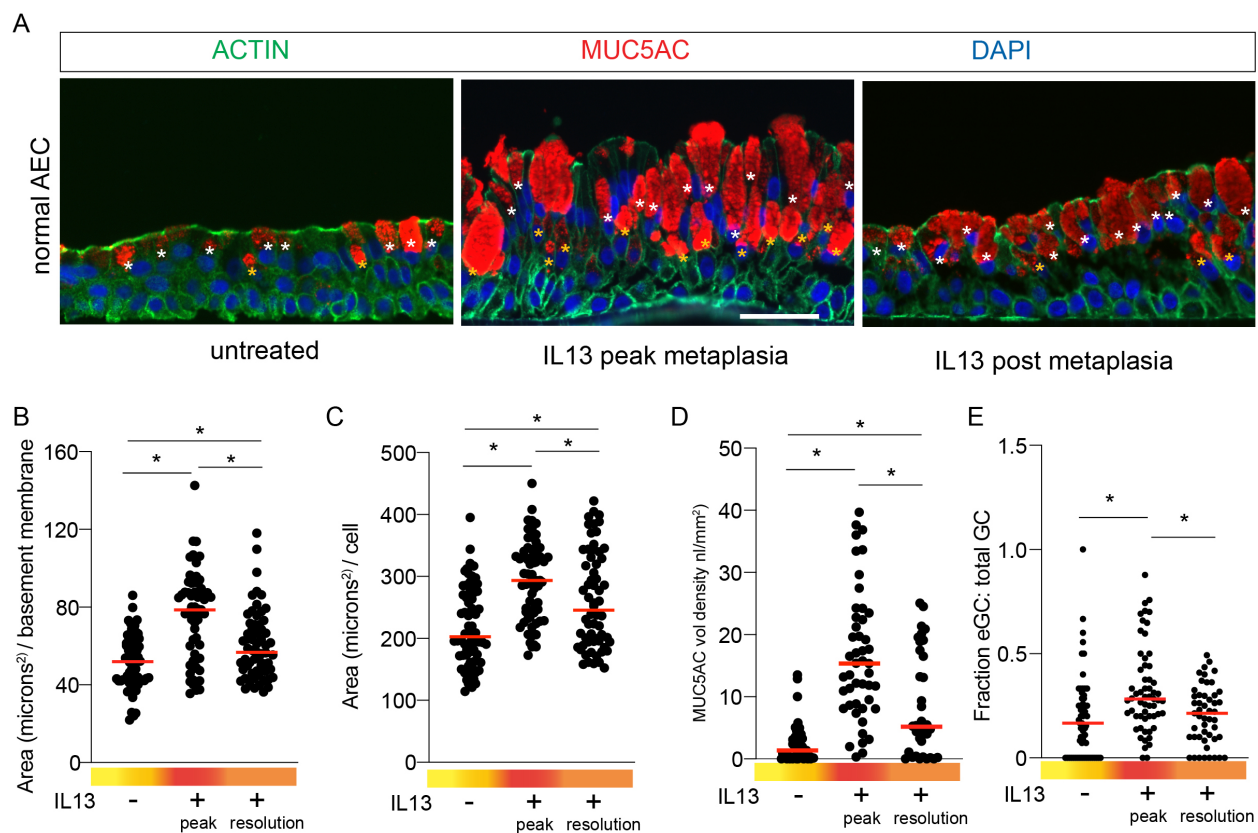

Supplemental Figure 1

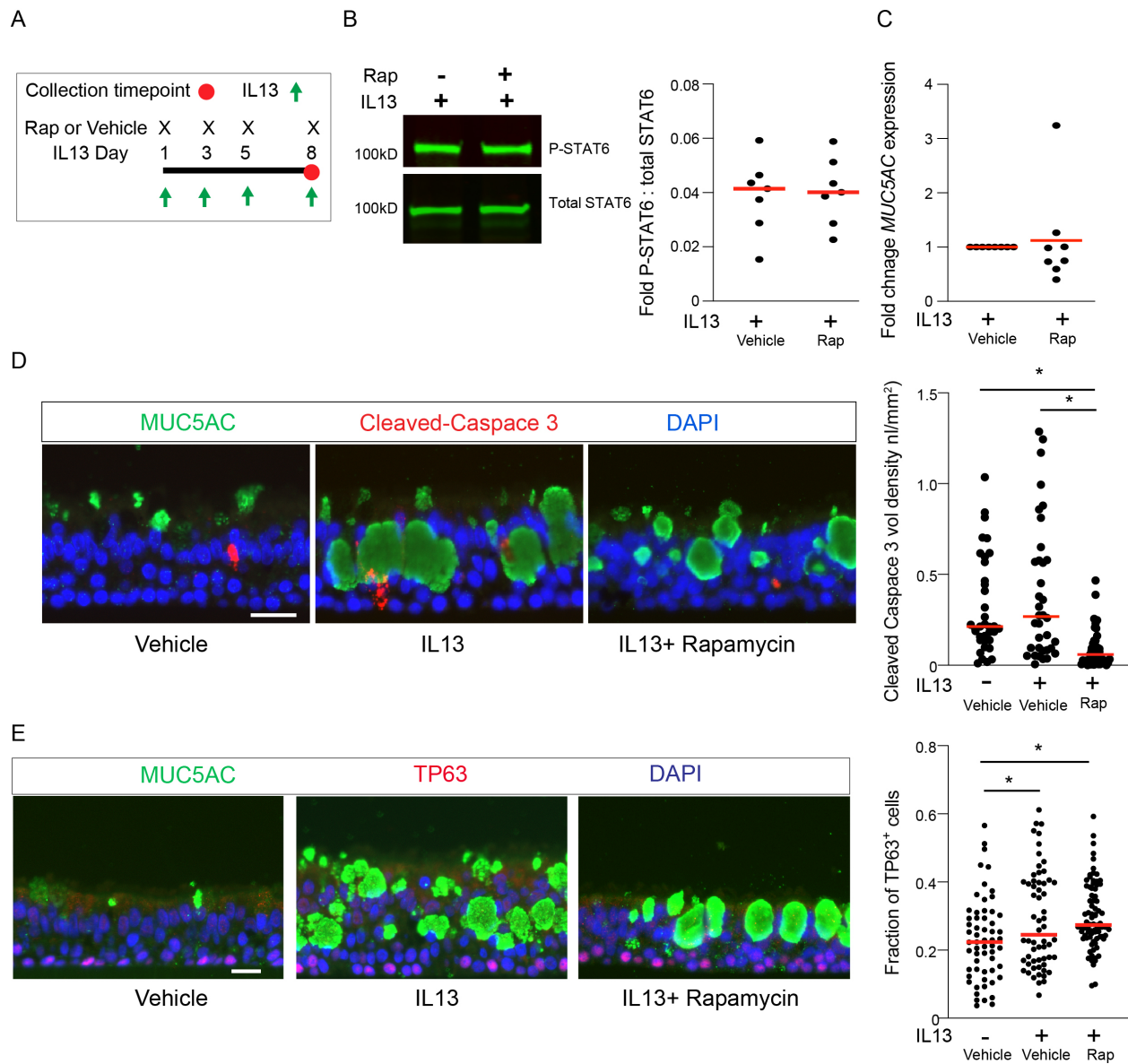

Supplemental Figure 2

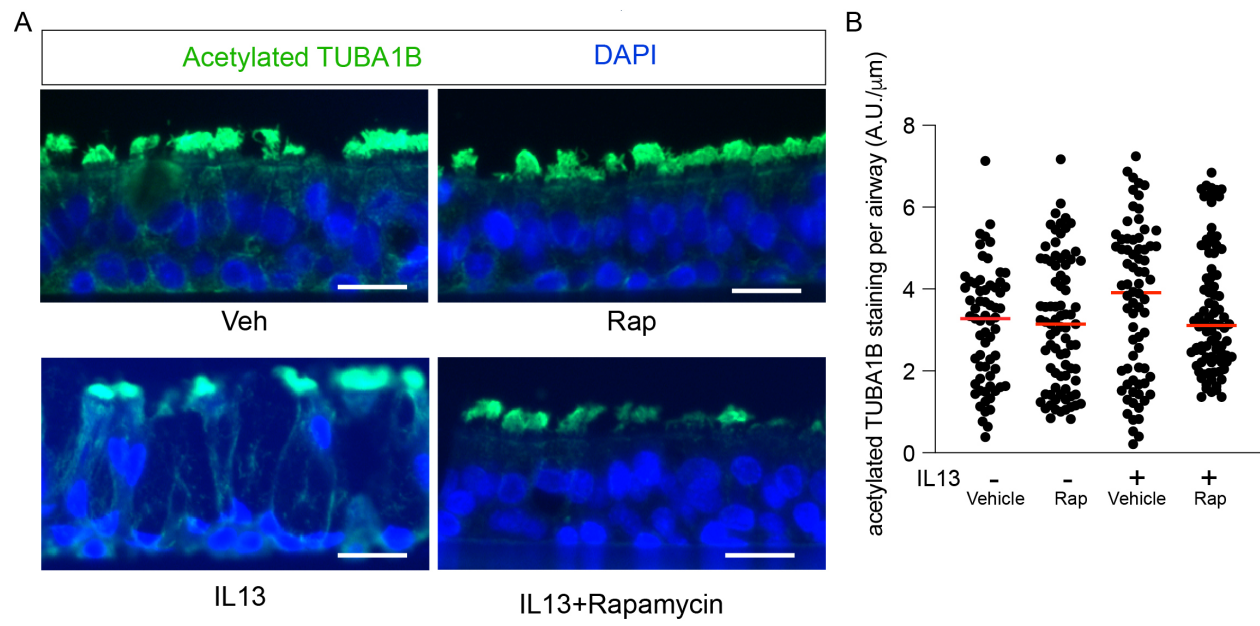

Supplemental Figure 3

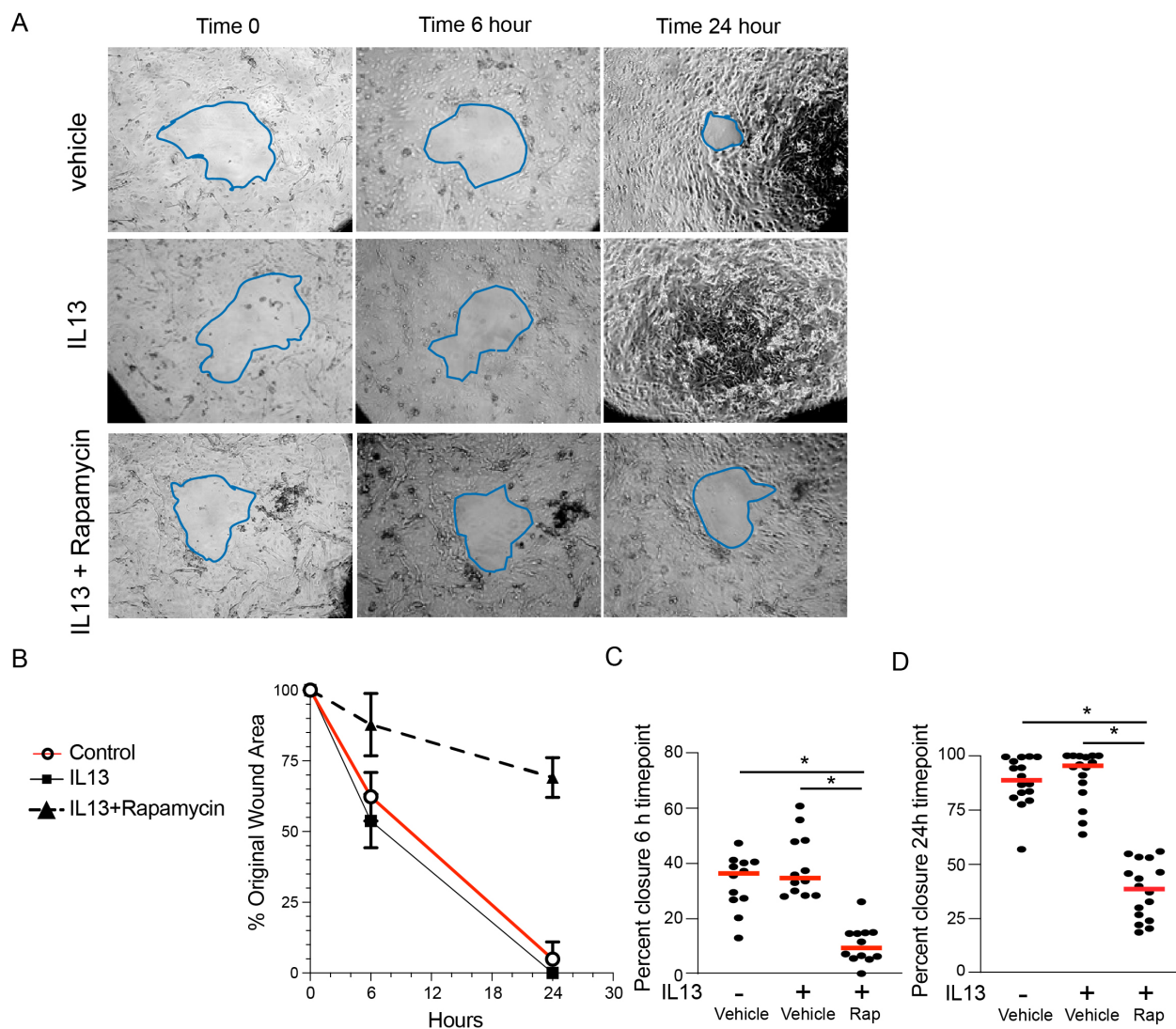

Supplemental Figure 4

Supplement: Supplemental figures 1-4 [file jciinsight-10-187904-s109.pdf]
