# Supplementary material for: MTOR signaling regulates the development of airway mucous cell metaplasia associated with severe asthma
Source: JCI Insight. 2025 May 29;10(13):e187904. doi: 10.1172/jci.insight.187904 (PMC12288895; doi:10.1172/jci.insight.187904)
Supplement: Unedited blot and gel images [file jciinsight-10-187904-s110.pdf]

Figure 3 total mTOR blots

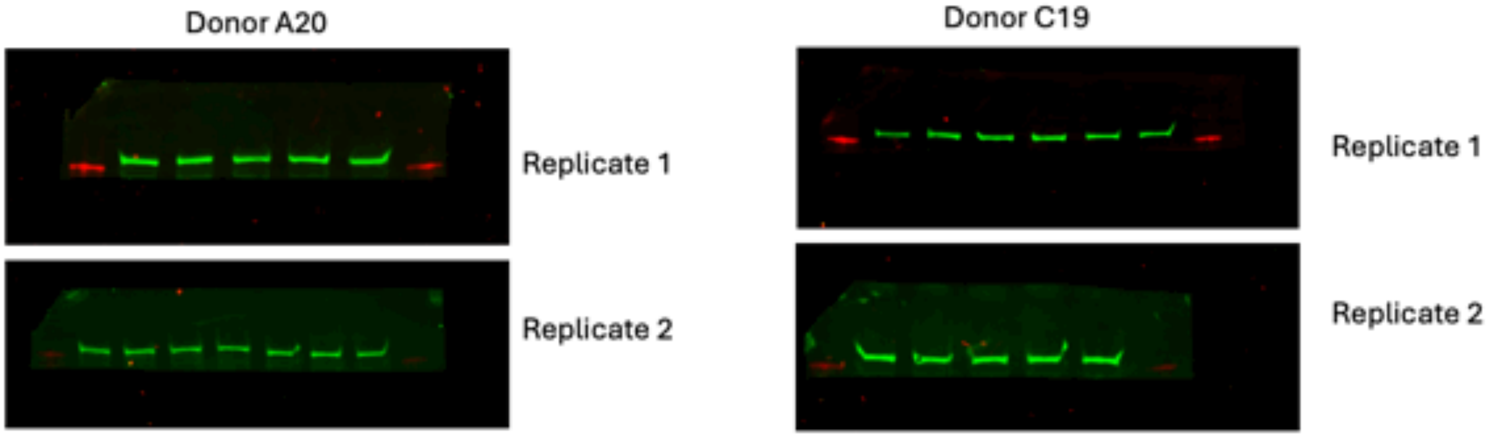

Figure 3 total mTOR blots continued

Donor B19

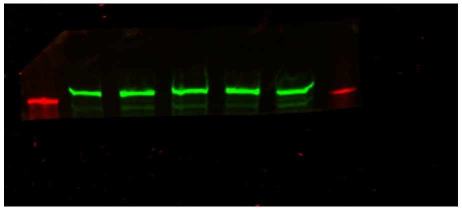

Replicate 1

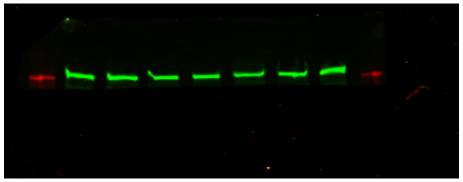

Replicate 2

Donor I-23

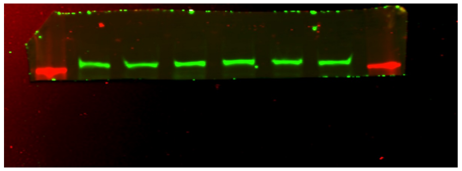

Replicates 1 and 2

Figure 3 Phospho RPS6 blots

A20 donor

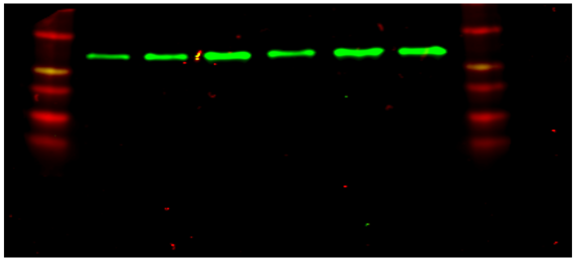

1st replicate

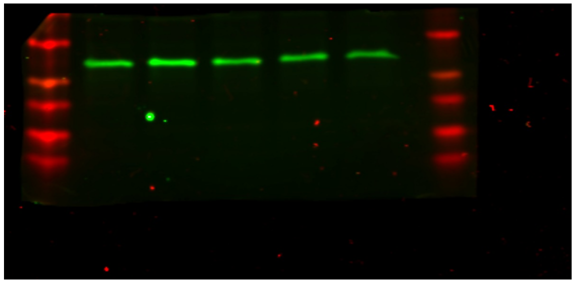

2nd replicate

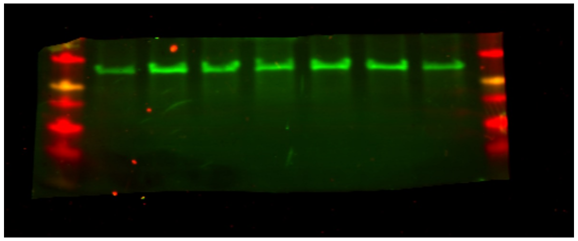

3rd replicate

B19 donor

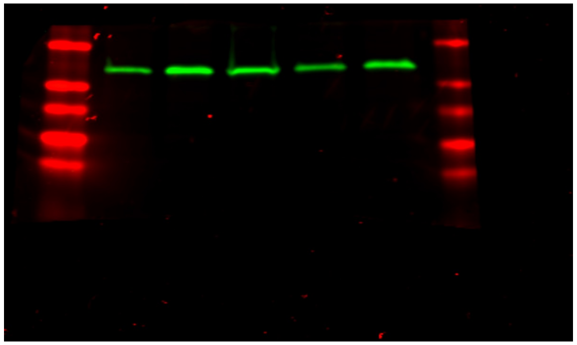

1st replicate

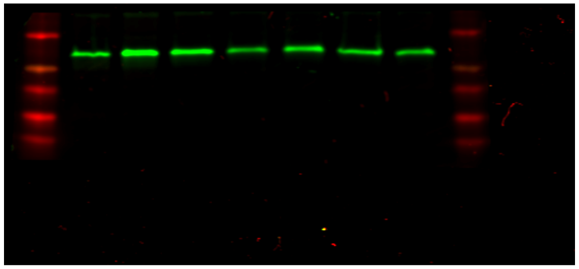

2nd replicate

Figure 3 Total RPS6 blots

A20 donor

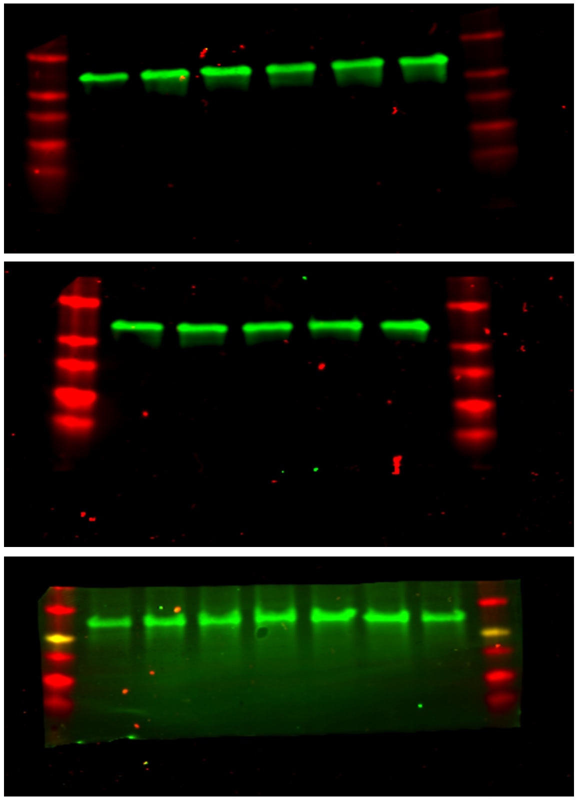

1st replicate

2nd replicate

3rd replicate

B19 donor

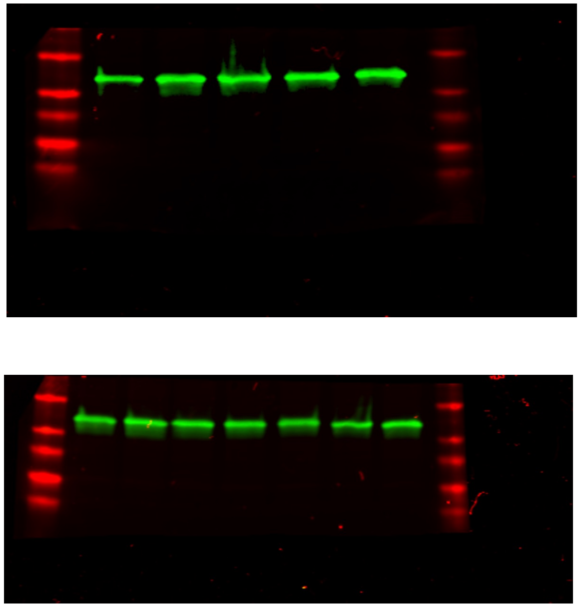

1st replicate

2nd replicate

Figure 3 Phospho RPS6 blots

C19 donor

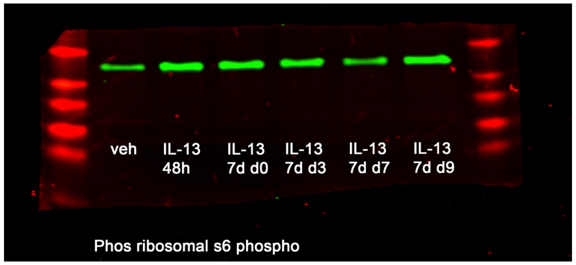

1st replicate

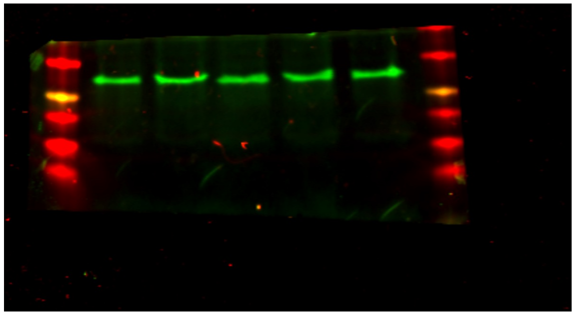

2nd replicate

I23 donor

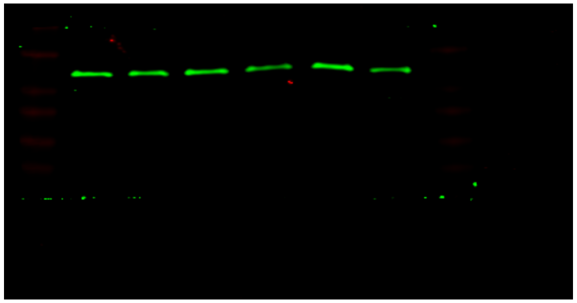

1st replicate

Figure 3 total RPS6 blots

C19 donor

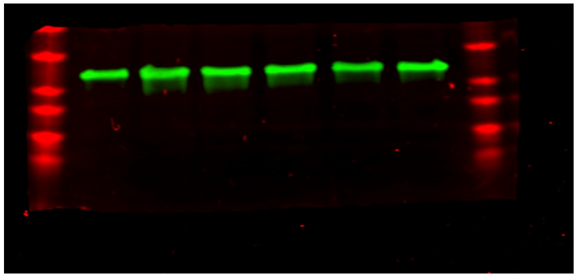

1st replicate

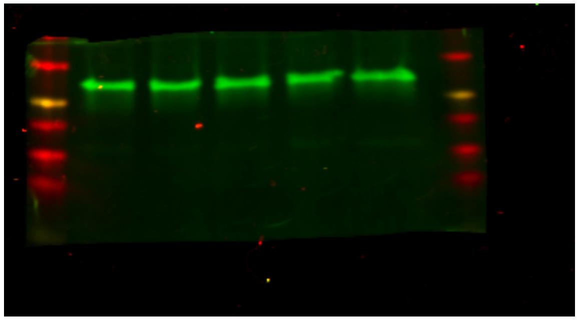

2nd replicate

I23 donor

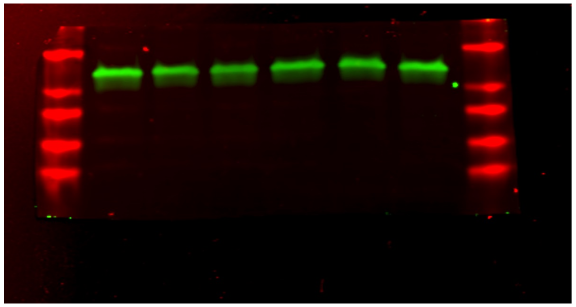

1st replicate

Figure 3 Phospho and total P70S6 blots

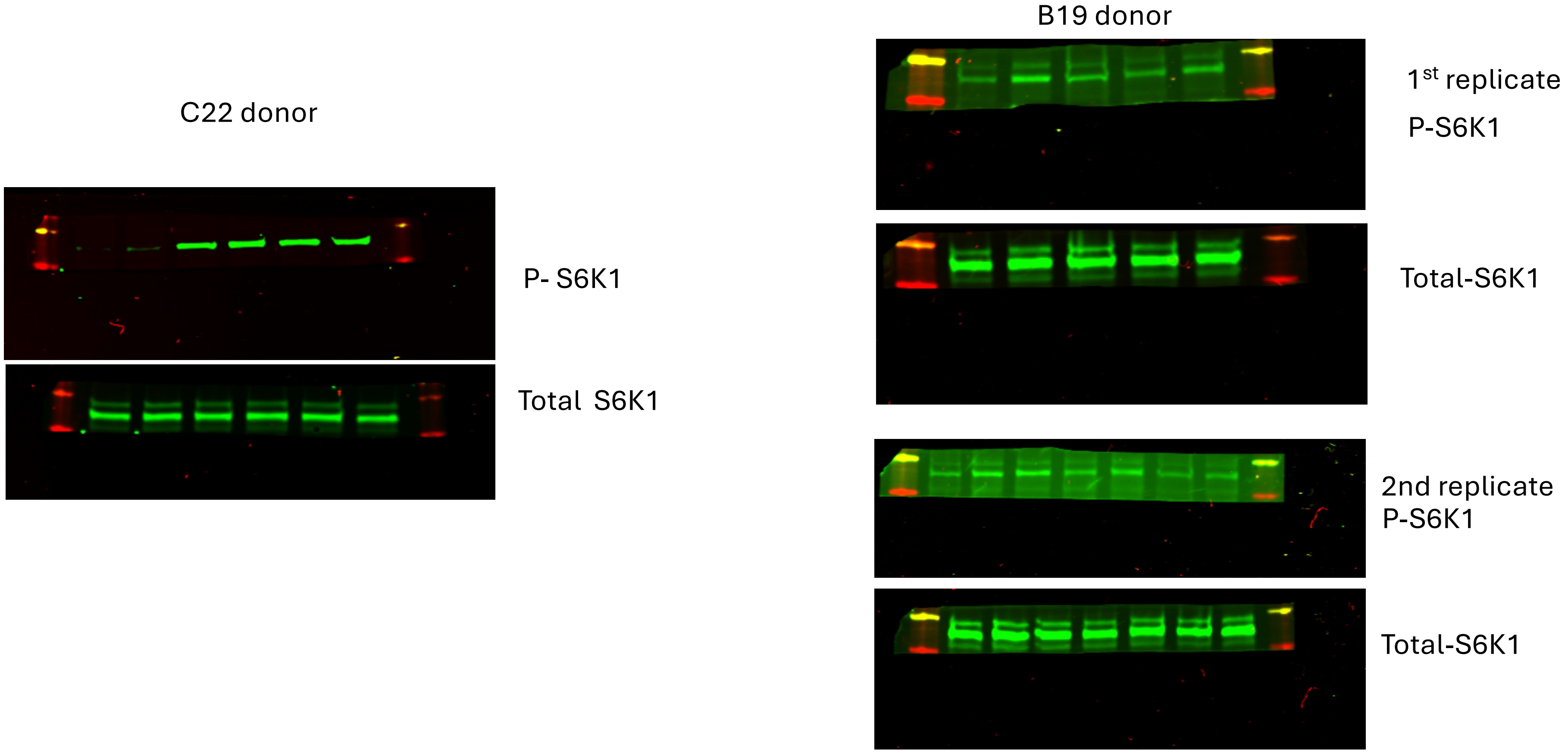

Figure 3 Phospho and total S6K1 blots

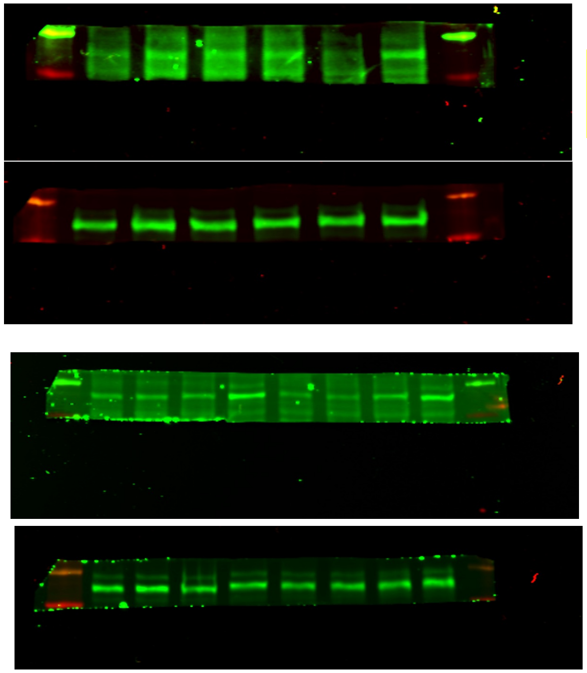

Figure 3 Phospho and total S6K1 blots

A20 donor

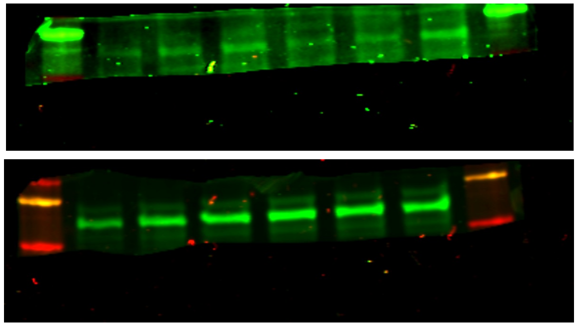

1st replicate
P-S6K1

1st replicate
Total S6K1

I-23 donor

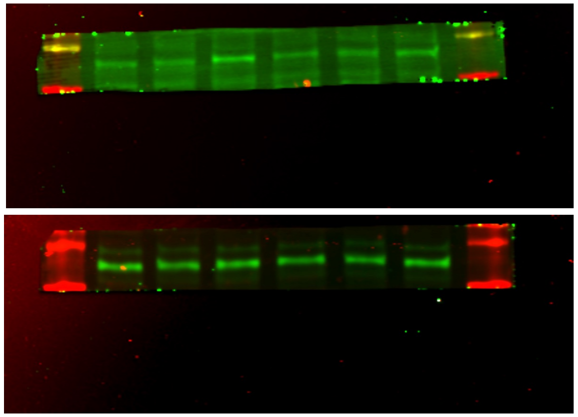

2 replicates
P-S6K1

2 replicates
Total S6K1

A20 donor

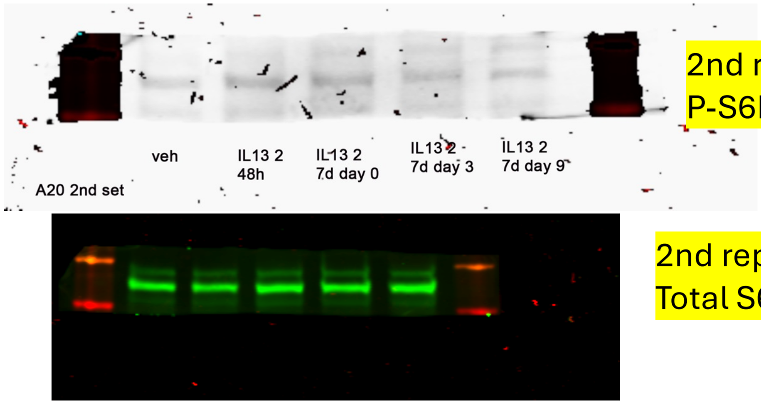

2nd replicate
P-S6K1

2nd replicate
Total S6K1

Figure 3 Phospho ULK1 blots

C21 donor 2nd experiment

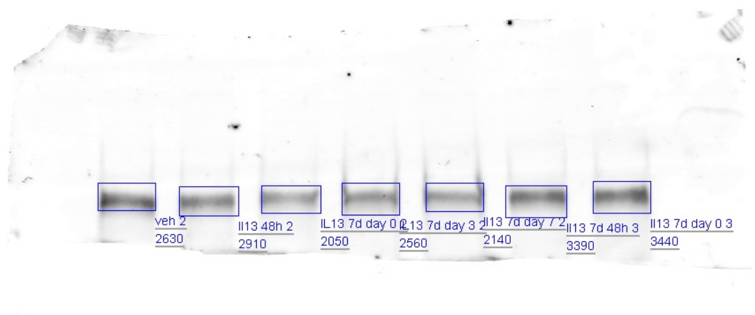

P- ULK1

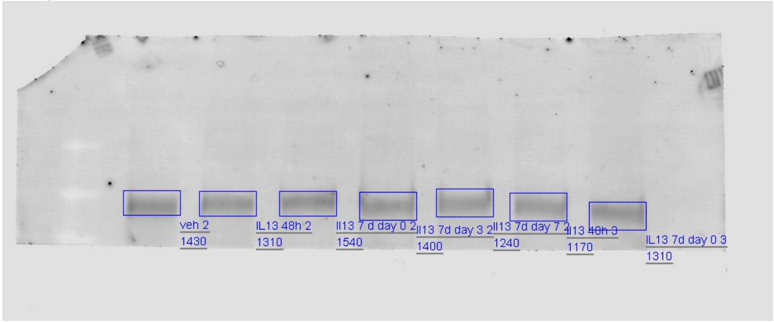

Total ULK1

Figure 3 Phospho and total ULK1 blots

C21 donor 1st experiment

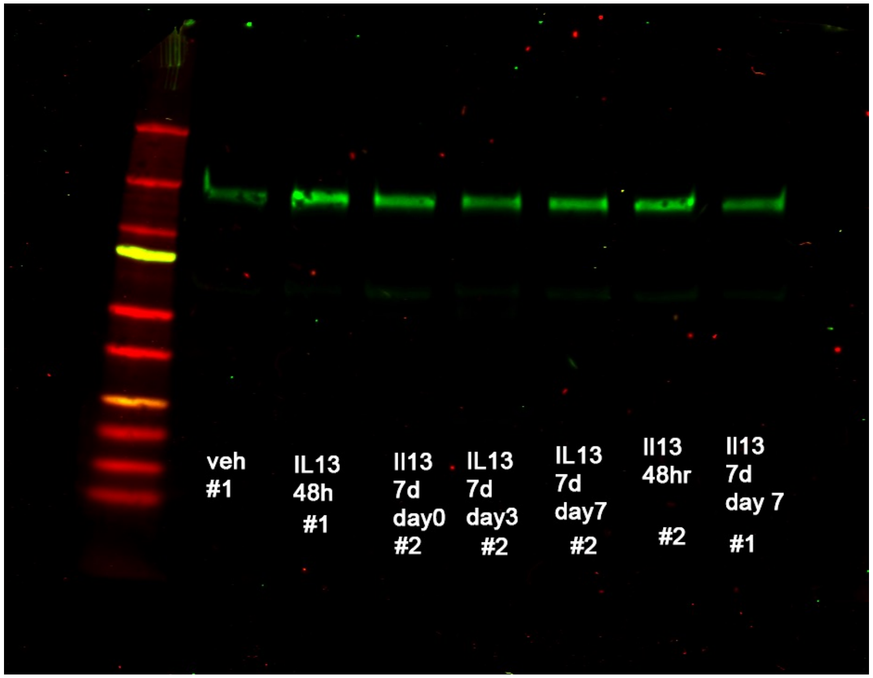

1st and 2nd replicates P-ULK1

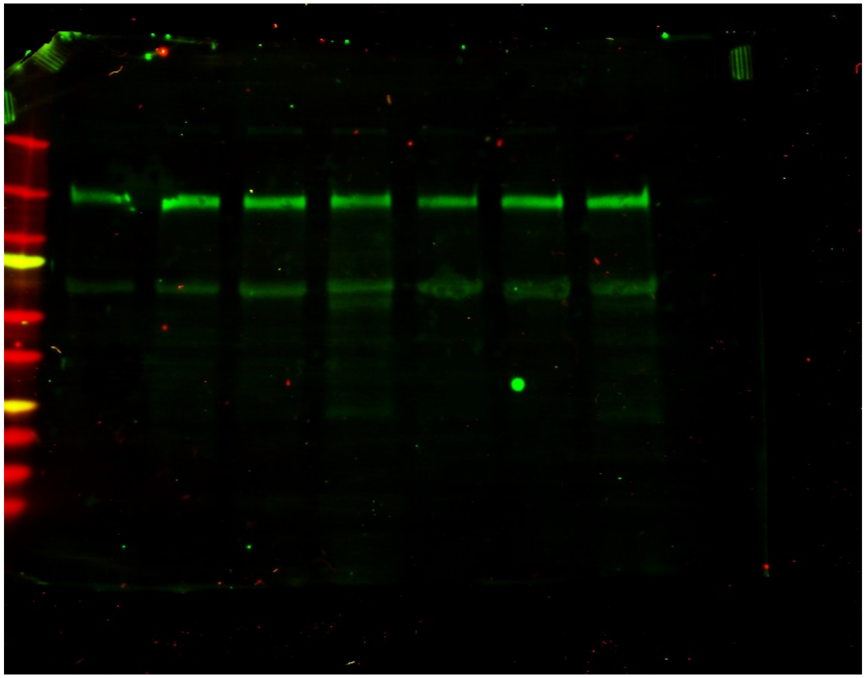

1st and 2nd replicates total ULK1

Figure 3 Phospho ULK1 blots

A20 donor 1st replicate

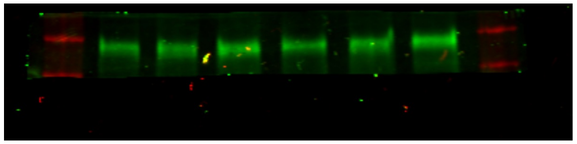

P- ULK1

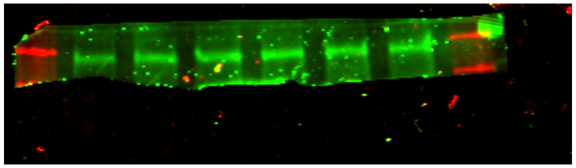

Total ULK1

A20 donor 2nd replicate

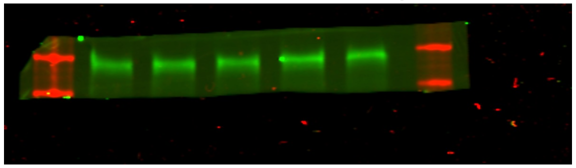

2nd replicate
P- ULK1

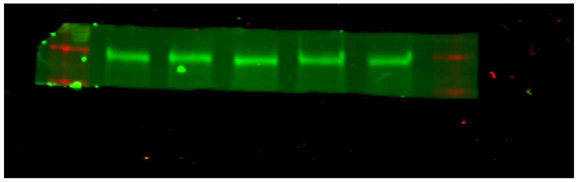

2nd replicate
Total ULK1

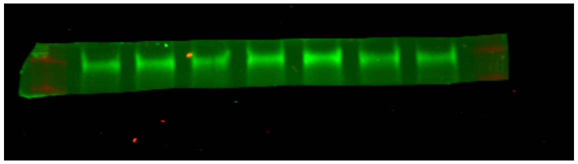

3rd replicate
P-ULK1

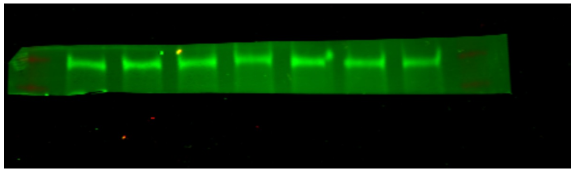

3rd replicate
Total ULK1

Figure 3 Phospho and total ULK1 blots

B19 donor

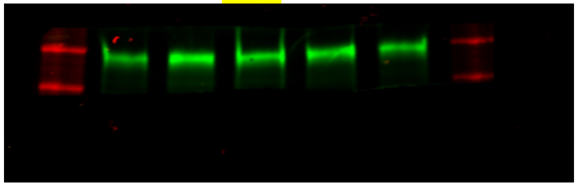

1st replicate
P-ULK1

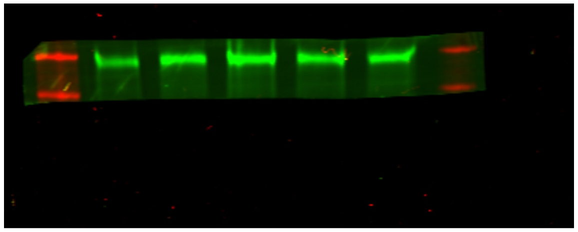

1st replicate
Total-ULK1

B19 donor

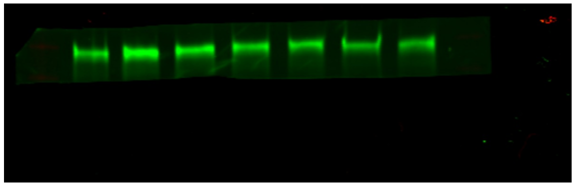

2nd replicate
P-ULK1

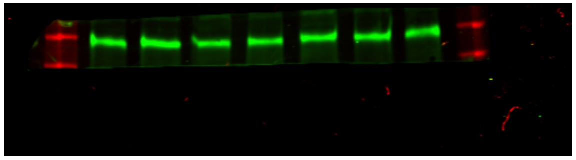

2nd replicate
total-ULK1

Figure 3 Phospho ULK1 blots

C19 donor

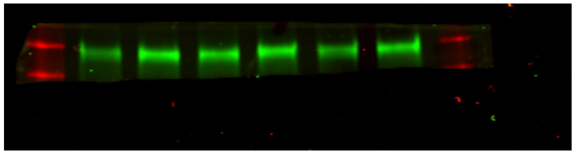

1st replicate
P-ULK

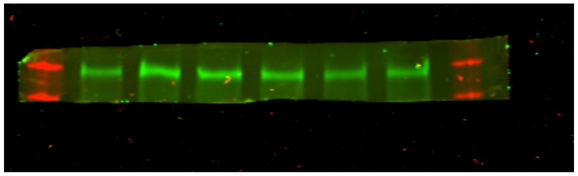

2nd replicate
total-ULK

C19 donor

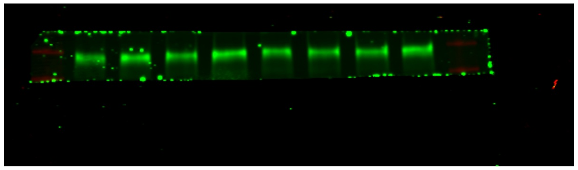

2nd replicate
P-ULK1

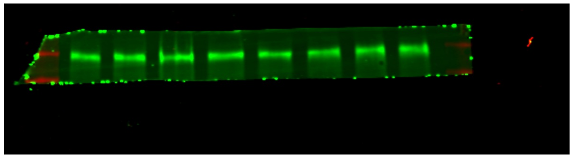

2nd replicate
total-ULK1

Figure 4 blots SQSTM1

A17 and A19 donors

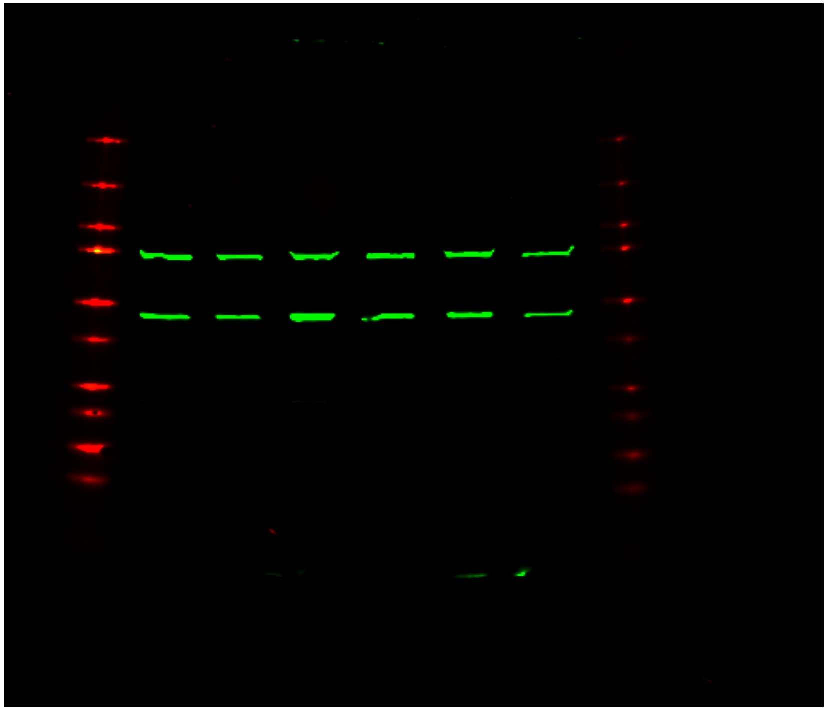

A17 and C22 donors

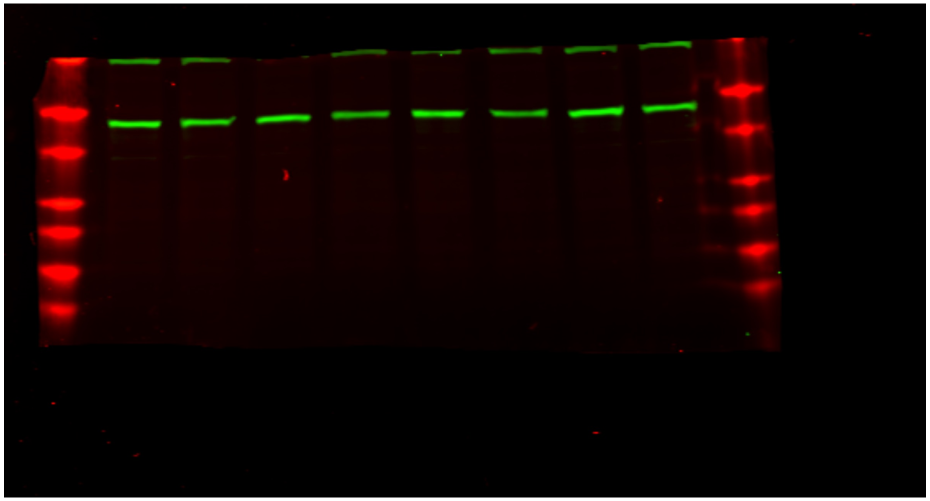

Figure 4 blots total and phosphor Rib S6

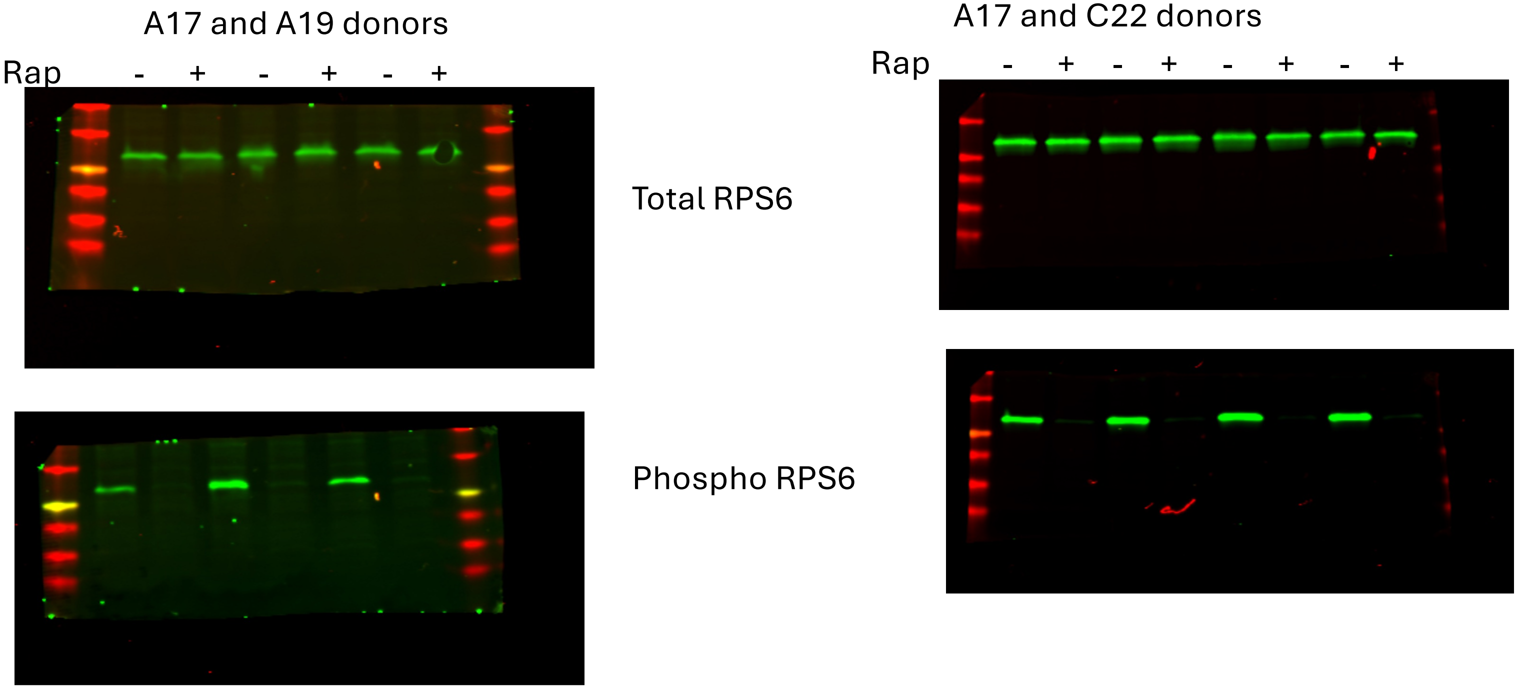

Figure 4A blots total and phospho PS6K1

A17 and A19 donors

Rap - + - + - +

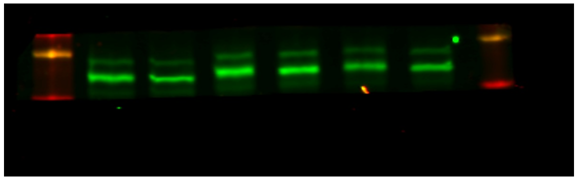

Total S6K1

Rap - +. - + - +

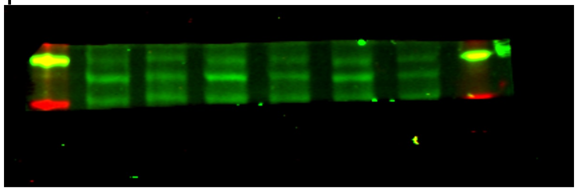

Phospho S6K1

A17 and C22 donors

Rap - + - + - + - +

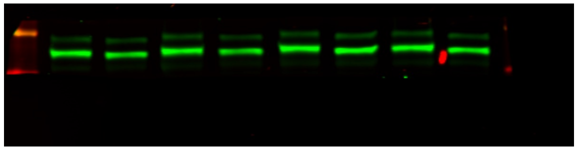

Total S6K1

Rap - + - + - + - +

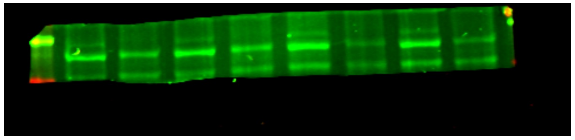

Phospho S6K1

Figure 5 MUC5AC blots

C22 and A17 donors

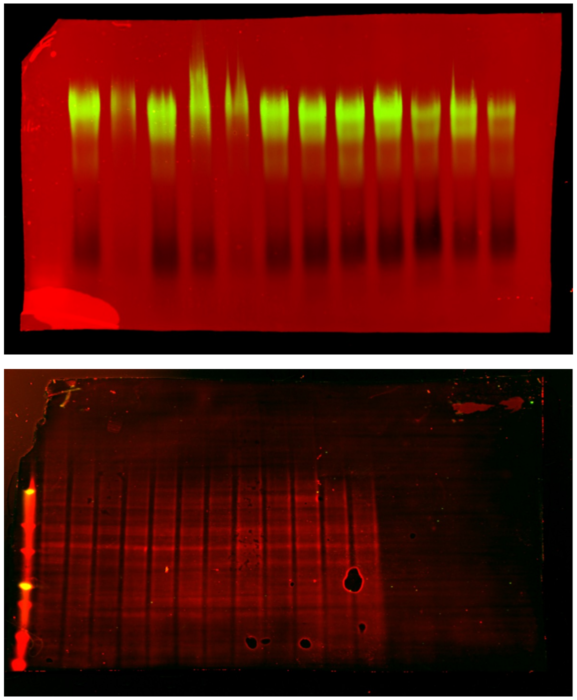

Total protein

A19 donor

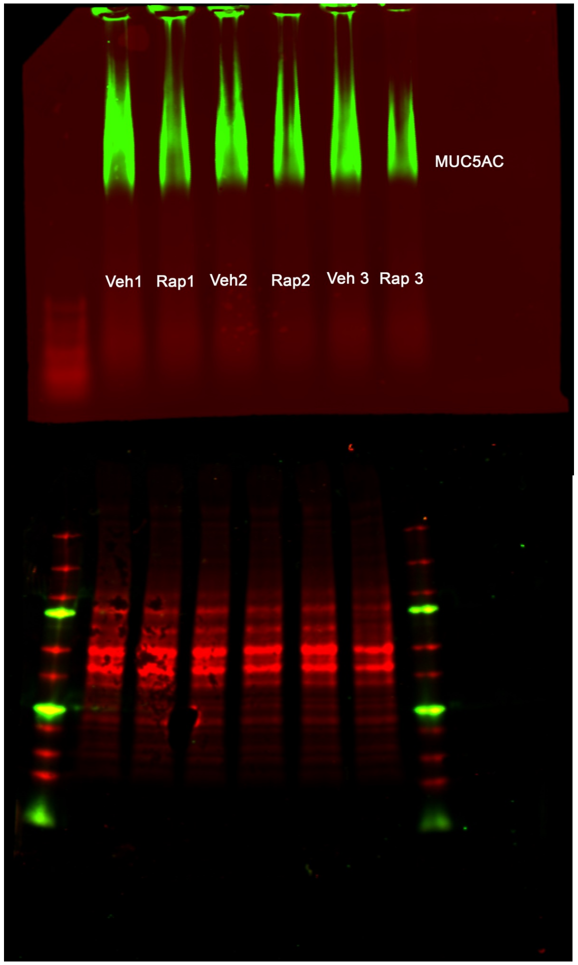

Total protein

Blots for Figure 8

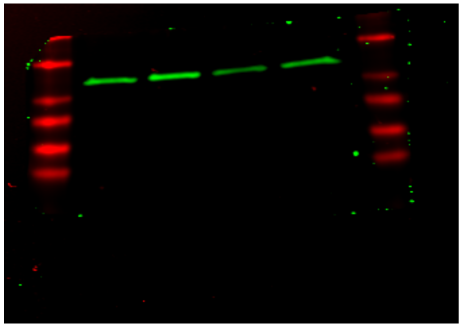

Phospho RPS6

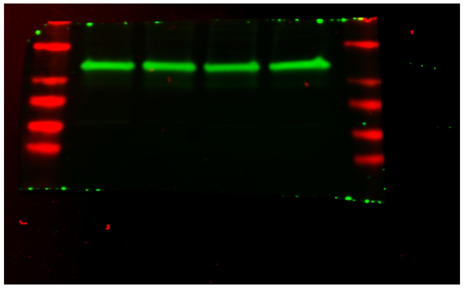

total RPS6

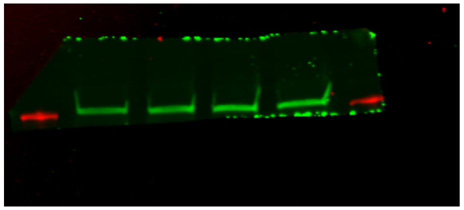

total mTOR
